# Depressive symptoms and the processing of unexpected social feedback: Differences in surprise levels, feedback acceptance, and “immunizing” cognition

**DOI:** 10.1371/journal.pone.0307035

**Published:** 2024-08-26

**Authors:** Lukas Kirchner, Winfried Rief, Lilly Müller, Hannah Buchwald, Kari Fuhrmann, Max Berg

**Affiliations:** Department of Psychology, Clinical Psychology and Psychotherapy, University of Marburg, Marburg, Germany; Public Library of Science, UNITED STATES OF AMERICA

## Abstract

Negative social expectations are a key symptom of depression. It has been suggested that individuals with depressive symptoms tend to maintain these expectations by devaluing new experiences that do not fit prior expectations. However, our understanding of the role of such “immunizing” cognition in response to unexpected social feedback in depression, as well as the cognitive mechanisms involved, remains limited. This study investigated the association between depressive symptoms and the cognitive processing of unexpected positive or negative social feedback using a novel, video-based approach featuring naturalistic social stimuli in a subclinical online sample (*N* = 155). We also examined how surprise levels, feedback acceptance and immunizing cognition relate to other cognitive processes, such as attributional style and rumination, using cross-sectional network analyses. Robust multiple linear regression analyses revealed that depressive symptoms were associated with higher surprise levels (*R*^2^_adj._ = .27), lower feedback acceptance (*R*^2^_adj._ = .19), and higher levels of immunizing cognition (*R*^*2*^_adj._ *=* .09) in response to unexpected positive social feedback, but only partially to unexpected negative social feedback. The network analysis suggested that self-efficacy expectations for coping with negative feelings and acceptance of positive social feedback had the strongest expected influence on the different cognitive processes. Our study highlights the challenges that individuals with depressive symptoms face in utilizing positive social feedback to modify negative expectations. For clinicians, our findings suggest the importance of promoting acceptance of positive social feedback, while simultaneously inhibiting immunizing cognition and avoiding the use of overly positive feedback.

## Introduction

Depression is characterized by serious impairments in social functioning that involve perception [1], cognition [2], mood [3], physiology [4], and behavior [5]. They affect both lower-level (e.g., working memory) [6] and higher-level processes (e.g., social decision-making) [7], and impair performance in both more simple (e.g., facial expression recognition) [8] and more complex (e.g., social problem-solving) [9] tasks. In many cases, it is therefore appropriate to view depression as a social disorder [10–13].

While it seems plausible that these deviations reflect formerly adaptive responses to “deleterious social environments” [14, p. 188] such as neglect and punishment during childhood [15], depressed individuals struggle to adapt to new social environments by updating their social experiences and behaviors accordingly to recover from these imprints [16].

A prominent example of this problem is negative social expectations (e.g., “Others will treat me badly.”) that tend to persist in individuals with depressive symptoms even when repeatedly facing contradictory evidence (e.g., receiving social support and appreciation from close relatives) [17–19]. The difficulty in updating expectations in response to corrective social experiences may contribute to depression’s self-reinforcing nature [20]. From this perspective, persistent negative expectations can shape future perceptions and behaviors in a way that eventually leads to a vicious cycle of pessimistic predictions, interpersonal maladaptation, negative interpersonal experiences, and even more pessimistic predictions for the future [21, 22].

Clinical theorizing and experimental studies suggest that negative expectations tend to persist in depression through undermining cognitions that emerge in response to contradictory, positive experiences (e.g., “Just because he was friendly to me once doesn’t mean he’ll be in the future.”) [19]. Although underlying cognitive mechanisms have not yet been fully elucidated, this phenomenon of becoming “immune” against new, surprising information (e.g., by reframing contradictory information) has been linked to cognitive processes frequently observed in depression, such as rumination or unfavorable attributional styles [23–25]. Accordingly, “cognitive immunization” has been introduced as a construct in the fields of depression and expectation research to describe all cognitive processes that “[…] invalidate the effect of positive, expectation violating experiences” [17, p. 5]. This includes re-appraising contradictory social experiences as “exceptions to the rule” or assigning low credibility to the experience itself [19].

Previous studies have linked cognitive immunization to the impaired updating of performance expectations [26–28]. They have also identified differences in cognitive immunization according to feedback valence [27], levels of surprise [26], and interventions while processing bogus performance feedback [28–30]. However, these differences have seldom been studied in the context of unexpected *social feedback* in depression [for laudable exceptions, see 26, 31]. Additionally, it is unclear which cognitive mechanisms determine cognitive immunization in the context of unexpected social feedback, their relative importance, and how they are interrelated [32, 33]. Shedding light on these cognitive underpinnings could have important implications for etiological concepts (e.g., relating different symptom domains), and psychological treatment (e.g., improving cognitive interventions).

### Research goals

The present study has three objectives. First, we test the hypothesis that depressive symptoms are linked to higher levels of immunizing cognition when individuals receive unexpected positive social feedback (H_1_). Second, we examine whether depressive symptoms are also associated with lower levels of immunizing cognition when individuals receive unexpected negative social feedback (H_2_). Third, as part of an exploratory investigation, we explore the interplay between depressive expectations, social feedback acceptance, immunizing cognition, levels of surprise, rumination, and attributional style. As it is difficult to measure immunizing cognition directly via self-reports, we chose to evaluate participants’ immunizing cognition by examining their thoughts following social feedback.

## Methods

Our study was conducted in accordance with the ethical guidelines of the German Psychological Association and was approved by the ethics committee of the psychology department at the University of Marburg (reference number: 2022-32k). We registered the study before collecting data at https://aspredicted.org/tj6tb.pdf. However, we made important changes to the protocol that are explained in detail below.

### Participants and data collection

*N* = 155 participants fully participated in our study. Data collection lasted from November 16, 2022, until November 30, 2022, and was conducted via the online research platforms *Prolific* (https://www.prolific.co/) and *Gorilla* (https://gorilla.sc/). Participants provided written informed consent before participating. They had to be at least 18 years old and fluent in German. To avoid stress for vulnerable individuals, we excluded participants with current suicidal thoughts or impulses. In so doing, we first administered the 18-item German version of the Brief Symptom Inventory (BSI-18) [34] to screen for suicidality. Participants reporting values higher than 2 on item 17 of the BSI-18 (“Idea to take your own life.”) were automatically excluded before entering the main part of the study. Participants who withdrew from participation before completing the study (i.e., exceeding the time limit of 3 hours) were also excluded.

Initially, we used information from Prolific’s “pre-screener questions” to distinguish between “depressed” and “healthy” participants, as specified in our pre-registration. “Depressed individuals” were required to have answered “Yes” to both of the following questions: “Do you have—or have you had—a diagnosed, ongoing mental health condition/illness/disease?” and “Do you experience depression?” Conversely, non-depressed individuals were required to answer “No” to these questions.

#### Changes to the registration

While reviewing the collected medical history information, we discovered that some of the participants’ responses differed from the pre-screener questions in Prolific. Despite initial indications, these participants did not always exhibit elevated levels of depression in their corresponding self-reports. To ensure a valid representation of the genuine level of current depressiveness, we decided to deviate from our protocol and relied on continuous depression scores from our self-report measures for our analyses. This adaptation changed our statistical approach and the way we operationalized our hypotheses. Originally, we planned to conduct simple group comparisons between the group with elevated depression levels and the group with lower depression levels on the dependent variables (DVs). However, we instead chose to perform multiple linear regression analyses using continuous indicators of depression as independent variables (IVs, see below). Nevertheless, we still included the group factor in our main analyses to control for potential differences between participants who reported a current or previous mental health issue.

Our initial power analysis suggested a sample size of 156 participants. This was based on the assumption of a small to medium effect (*d* = .40), with an alpha level of α = .05, and a target power of 80%. However, we observed significant increases in power after modifying our statistical approach. A post hoc analysis revealed a power of 92%, assuming three predictor variables, a sample size of *N* = 155, an alpha level of α = .05, and a determination coefficient of *R*^*2*^_adj_ *=* .09.

Another change concerns the selection of indicators for the assessment of immunizing cognition. The original plan was to use the average feedback acceptance rating (see below) as the primary dependent variable (DV, see https://aspredicted.org/tj6tb.pdf). Although the results for these ratings were consistent with our registered hypotheses (see manipulation checks), we decided to analyze these ratings separately in the results section and our second registered indicator of immunizing cognition instead (see below). We believe this is suitable because a lack of feedback acceptance indicates only one potential outcome of immunizing cognition, rather than the presence of immunizing thoughts itself.

### Procedure and material

To investigate the cognitive processing of unexpected social feedback in a thorough and ecologically valid manner, we took a novel approach that aimed at measuring explicit and implicit cognitive processes. First, we created a pool of 21 negative and 21 positive social feedback statements, which were loosely based on the social expectations contained in the “Social Rejection” and “Social Support” subscales of the DES (S2 Appendix). In the first pilot test, we asked ten clinical psychologists to rate the pool: For each negative feedback statement, the raters indicated on a 6-point Likert scale how typical it would be for depressed patients to expect such feedback from their social environment (0: “not typical at all” vs. 5: “very typical”), and how problematic it would be to expect such feedback (0: “not problematic at all” vs. 5: “very problematic”). For each positive feedback statement, the raters indicated on a 6-point Likert scale how surprised patients with depression would be receiving such feedback (0: “not surprised at all” vs. 5: “very surprised”) and how helpful the aspiring therapists consider this feedback with respect to the patients’ mental health (0: “not helpful at all” vs. 5: “very helpful”).

**Fig 1:**
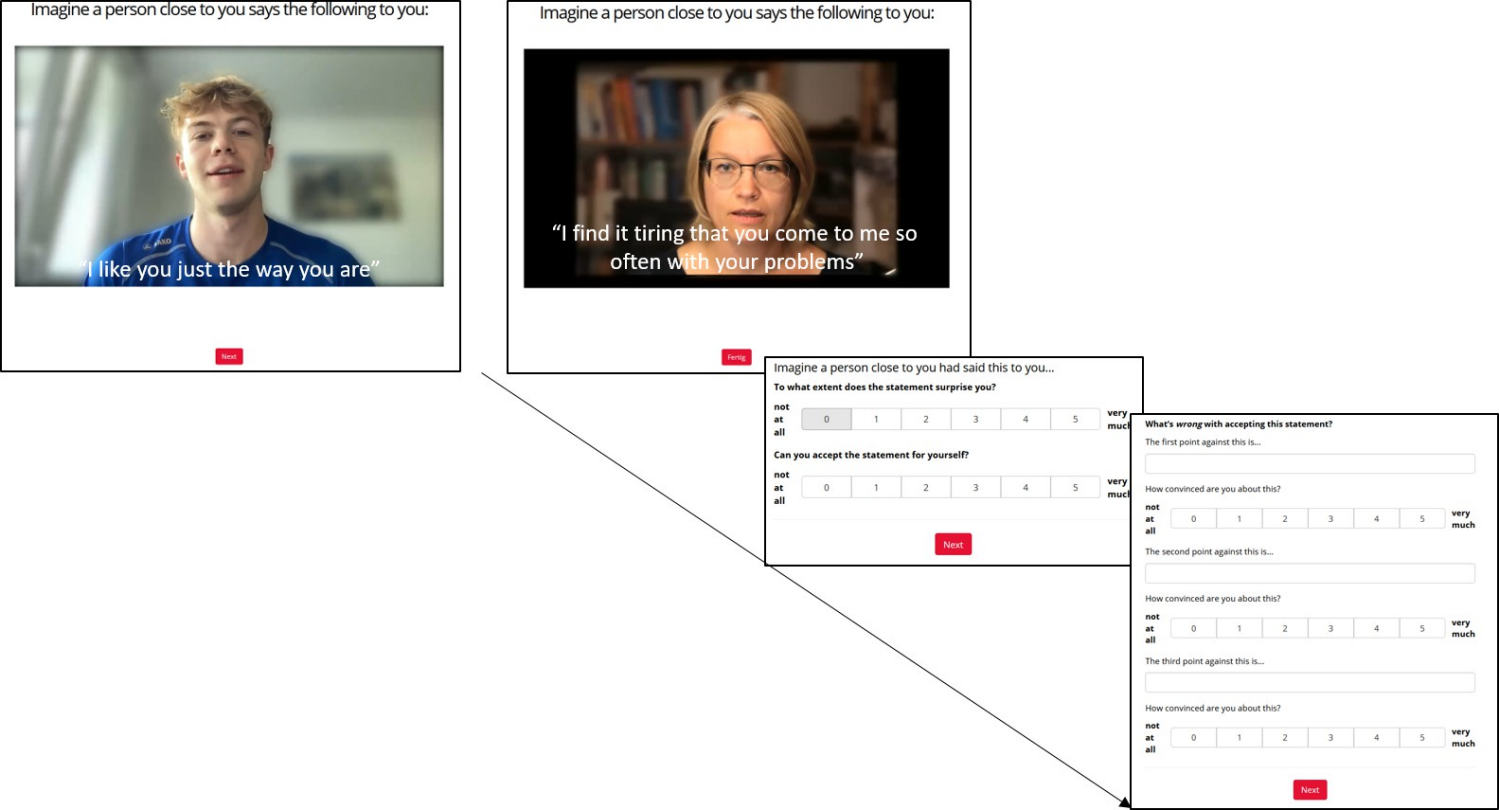
Trial sequence. Left: Excerpt from a positive video stimulus. Right: Excerpt from a negative video stimulus.

Relying on this information, we selected three positive and three negative statements as stimulus material for our study. These statements had been rated as above-average “typical” for people with depression, or, in case of the positive statements, as above-average “surprising” for people with depression (for detailed information regarding statement selection, see S2 Appendix). In succession, we created short video messages with different people presenting the selected statements in a webcam-conference setting. We balanced speakers’ age (20 to 70 years) and gender between the negative and positive feedback statements. When recording the video sequences, we blurred the speakers’ backgrounds (Fig 1).

In the second pilot test, we recruited 20 healthy participants on Prolific who had negated the pre-screening questions in Prolific. We aimed to test the effectiveness of our procedure, including our instructions and the manipulation, and explore whether presenting the selected statements as videos or audios had revealed any impact. Based on those results, we decided to use video rather than audio stimuli in our main study (S3 Appendix).

Both pilot tests allowed us to refine our stimulus selection, ensuring that we would indeed capture the (partially implicit) cognitive processing of unexpected social feedback.

**Fig 2:**
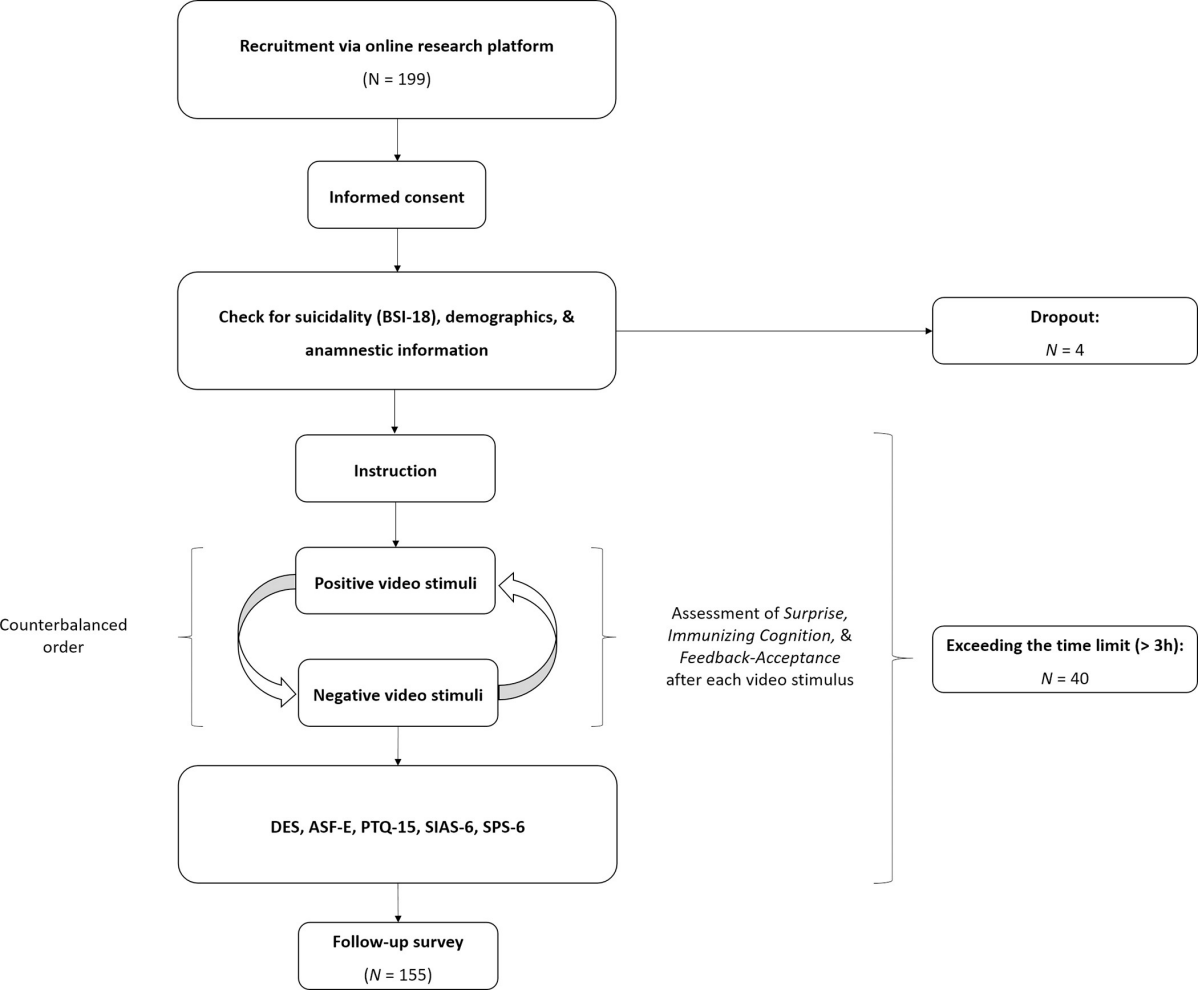
Study design. This flowchart illustrates the study’s design and procedure, including the recruitment process and dropout rates.

In the main study, we began by obtaining informed consent and assessing suicidality, then recorded the remaining items of the BSI-18. After that, we collected demographic and anamnestic data. Before presenting the first video stimulus, we asked participants to imagine a person from their social circle speaking the content. They were then allowed to start the video, absorb its content, and then share their surprise, feedback acceptance, and any thoughts the video provoked (refer to Fig 1 and below). This process was repeated for each video stimulus. At the end of the study, we administered the remaining questionnaires and conducted a follow-up survey to assess any potential stress caused by the video stimuli. Fig 2 provides an overview of the study’s design.

### Measures

#### Demographics and anamnestic information

We collected data on the following variables using a self-report questionnaire: *Gender* (“male” vs. “female” vs. “diverse”), *Citizenship* (“German” vs. “other” vs. “mixed”), *Legal Status* (“unmarried” vs. “married” vs. “divorced” vs. “widowed” vs. “registered partnership”), *Relationship Status* (“single” vs. “partnership”), *Education* (“major school diploma” vs. “secondary school diploma” vs. “high school diploma”), *Vocation* (“none/not completed” vs. “completed” vs. “Bachelor’s degree” vs. “Master’s degree” vs. “PhD”), *Employment* (“full-time” vs. “part-time” vs. “not employed”, *Migration* (“immigrant” vs. “no immigrant”, *Age* (in years), *Weekly Working Time* (in hours), *Household Members* (number), and *Net Monthly Household Income* (in Euro). Additionally, we collected anamnestic information on the following variables: *Anamnestic Mental Health Issue* (“no mental health issue” vs. “mental health issue”), *Diagnosis of Major Depression* (“never” vs. “currently or in the past”, *Outpatient Treatments* (“never” vs. “currently or in the past”), *Inpatient Treatments* (“never” vs. “currently or in the past”, *Medication* (“never” vs. “currently or in the past”), and *Disorder Onset* (age in years).

#### Depressive symptoms and general mental health

We used the short German version of the Brief Symptom Inventory (BSI-18) [34] to assess symptoms of *Depression*, *Anxiety*, and *Somatization*. The subscales “Anxiety” and “Somatization” were assessed only for descriptive purposes. Each subscale comprises six symptoms (e.g., “feelings of loneliness”) rated on a 5-point Likert scale ranging from 0 (“not at all”) to 4 (“very strong”). Participants indicated how much they had suffered from these symptoms “in the past seven days”. Higher composite scores indicate higher levels of depression, anxiety, or somatization. The internal consistency indices [35] were as follows: ω = .90 (“Depression” subscale), ω = .83 (“Anxiety” subscale), ω = .77 (“Somatization” subscale), and ω = .92 (global scale).

We used the German version of the Depressive Expectations Scale (DES) [36] to evaluate levels of *Depressive Expectations*. The DES consists of 25 expectations that are typical for people with depression, and is divided into four subscales (“Social Rejection”, “Social Support”, “Mood Regulation”, and “Ability to Perform”). Each expectation is rated on a 5-point Likert-type scale ranging from 1 (“I disagree”) to 5 (“I agree”). While some items need to be inverted before computing the composite scores, higher composite scores indicate more expectations typical of depression (i.e., expectations of social rejection, mood regulation ability, ability to perform, and lower social support). The global scale showed high internal consistency (ω = .91). The subscales also demonstrated good to acceptable internal consistency: ω = .83 for the “Social Rejection” subscale, ω = .82 for the “Social Support” subscale, ω = .78 for the “Mood Regulation” subscale. Note that we could not calculate ω for the “Ability to Perform” subscale as it consists only of 2 items.

#### Surprise after social feedback

After each video stimulus that incorporated positive or negative social feedback (Fig 2), we measured *Surprise* using one-item 6-point Likert scales (“To what extent does the statement surprise you?”) ranging from 0 (“not at all”) to 5 (“very much”). We calculated *Surprise after Positive Social Feedback* by taking the mean of these ratings for each video stimulus with positive feedback (e.g., “I like you just the way you are.”). Similarly, *Surprise after Negative Social Feedback* was obtained by taking the mean of these ratings for each video stimulus with negative feedback (e.g., “It’s exhausting that you always come to me to talk about your problems.”). Higher values indicate greater surprise after social feedback. The internal consistencies ranged from ω = .76 (“Surprise after Positive Social Feedback”) to ω = .68 (“Surprise after Negative Social Feedback”).

#### Feedback acceptance

Following the social feedback, we used one-item 6-point Likert scales (“Can you accept this statement for yourself?“) after each video stimulus, ranging from 0 (“not at all”) to 5 (“very much”). The mean of these ratings served as a measure of *Feedback Acceptance*, calculated separately for positive (*Feedback Acceptance after Positive Social Feedback*) and negative social feedback (*Feedback Acceptance after Negative Social Feedback*). Due to only three ratings being obtained for each type of feedback, the internal consistencies were rather poor (i.e., ω = .67 for positive feedback and ω = .56 for negative feedback).

#### Immunizing cognition

Participants were asked to write up to three thoughts after each video stimulus regarding the question, “What’s wrong with accepting this statement?”. They then rated their conviction level for each thought on a 6-point Likert scale ranging from 0 (“not at all”) to 5 (“very much”). The first author rated participants’ thoughts using a codebook based on examples of cognitive immunization from the literature (S1 Appendix), labeling them as either 1 (“immunizing”) or 0 (“not immunizing”) and multiplying this score with the corresponding conviction rating. The resulting scores were averaged for each participant across all thoughts and video stimuli, but separately for positive and negative video stimuli. Higher scores indicate higher levels of *Immunizing Cognition* for positive versus negative social feedback. The internal consistency was ω = .73 for “Immunizing Cognition” after positive social feedback, and ω = .54 for “Immunizing Cognition” after negative social feedback.

To assess inter-rater reliability, a master’s student rated 10.29% of participants’ thoughts again using the same codebook. The raters’ agreement was 75.00%, and Cohen’s Kappa indicated moderate interrater reliability (κ = 0.49) [37].

#### Attributional style

To assess the *Attributional Style*, we adapted parts of the German Attributional Style Questionnaire for Adults (ASFE-E) [38]. The ASF-E contains 16 descriptions of positive and negative situations (e.g., “You meet a friend who compliments you.”). Participants are asked to identify one main cause that explains what happened in each situation (e.g., “What is the main cause for the compliment?”). Participants are then asked to rate how much the named cause was internal to themselves (1: “The main reason for a friend’s compliment is completely due to other people or circumstances” vs. 7: “The main reason for a friend’s compliment lies entirely within myself.”), and how stable (1: “The main reason for a friend’s compliment will never influence whether a friend compliments me again in the future.” vs. 7: “The main reason for a friend’s compliment will continue to influence whether or not a friend compliments me in the future.”) or global (1: “The main reason for a friend’s compliment only influences whether or not a friend compliments me” vs. 7: “The main reason for a friend’s compliment also positively influences many other areas of my life”) the cause appeared on 7-point Likert scales (always two ratings for each dimension).

As we were particularly interested in participants’ attribution style in social situations, we only used the eight items of the ASF-E that dealt with positive or negative social content, discarding all the other items. Higher composite scores on the “Internality,” “Stability,” and “Globalism” subscales for negative social situations indicate a more negative attributional style, while for positive social situations, they indicate a more positive attribution style.

For the negative social scenarios, internal consistencies were ω = .77 for the “Internality” subscale, ω = .83 for the “Stability” subscale, and ω = .88 for the “Globalism” subscale at the subscale level, and ω = .93 for the overall scale. For the positive social scenarios, on the other hand, internal consistencies showed ω = .63 for the “Internality” subscale, ω = .50 for the “Stability” subscale, and ω = .77 for the “Globalism” subscale, and were ω = .83 for the overall scale.

#### Rumination

We used the 15-item German version of the Perseverative Thinking Questionnaire (PTQ-15) [39] to assess rumination. The PTQ-15 consists of 15 statements about one’s thoughts or thought processes (e.g., “The same thoughts keep going through my mind again and again.”). Respondents rated these items on a 5-point Likert scale ranging from 0 (“never”) to 4 (“almost always”). Note that higher composite scores on the PTQ-15 indicate higher levels of rumination. McDonald’s ω for the PTQ-15 was .97.

#### Symptoms of social phobia and social anxiety

We used the 6-item German versions of the Social Anxiety Scale (SIAS-6) and Social Phobia Scale (SPS-6) to assess symptoms of social anxiety and social phobia [40]. Each scale comprises six items that describe typical symptoms of social anxiety (e.g., “I feel tense if I am alone with just one person.”) or social phobia (e.g., “I get nervous that people are staring at me as I walk down the street.“). Participants were instructed to rate each symptom on a 5-point Likert scale ranging from 0 (“not at all characteristic of me”) to 4 (“extremely characteristic or true of me”). Higher composite scores indicate higher levels of social anxiety or social phobia. The internal consistencies were ω = .85 (SIAS-6) and ω = .92 (SPS-6).

#### Distress due to participation

We assessed an indicator of *Distress due to Participation* by combining three ratings on how burdened, stressed, and depressed participants felt due to their participation. Participants responded on a 6-point Likert scale ranging from 0 (“not at all”) to 5 (“very much”) for all three ratings, with higher values indicating higher levels of *Distress due to Participation*. The internal consistency of the ratings was ω = .85.

### Data preparation and statistical analyses

Of the *N* = 155 participants who met our study’s inclusion criteria and fully participated in our study, eight participants (5.16%) were identified as potential outliers through box-plot inspection (values > ± 1.5 IQR). In addition, 20 participants (12.90%) reported experiencing technical problems during participation in the follow-up survey, such as being unable to play one of the video stimuli. However, sensitivity analyses showed that excluding these cases did not substantially alter the result pattern of our main analyses, including the magnitude and direction of the effects of interest. Therefore, we chose to base our analyses on the entire sample.

To provide manipulation checks, we performed multiple linear regression analyses with our indicators of *Depression*, *Depressive Expectations*, and *Anamnestic Mental Health Issue* (i.e., the group factor was included because of our initial recruitment strategy) as IVs and our indicators *Surprise* after social feedback and *Feedback Acceptance* as the DVs. Similarly, we conducted separate multiple linear regression analyses with the same IVs and our indicator of *Immunizing Cognition* as DV to test our hypotheses. In the second step, we included indicators of *Social Anxiety* and *Social Phobia* in the latter analyses to test whether the effects persisted when we controlled for these two variables.

We checked assumptions using collinearity diagnostics, case-specific diagnostics, QQ-plots, and residual plots. Cook’s distance did not reveal any outliers in any case (all values < 1). Since assumptions of homoscedasticity or normality were violated in some cases, we used robust parameter estimation (5000 bootstrap samples).

Finally, we performed an exploratory network analysis to examine the cognitive processing of unexpected social feedback. Specifically, we investigated the interaction and relative importance of our indicators of *Depression*, *Surprise*, *Feedback Acceptance*, *Cognitive Immunization*, *Attributional Style*, and *Rumination*. We estimated a partial correlation network and used LASSO regularization (Least Absolute Shrinkage and Selection Operator) [41] to eliminate unreliable edges based on the Extended Bayesian Information Criterion (EBIC) [42], with γ = 0.5. To estimate edge centrality, we calculated one-step expected influence (EI), a measure shown to better reflect strength centrality in psychological networks compared to traditional indicators [43]. EI is defined as the summed weight of a given node’s edges shared with the remaining nodes in the network [43].

We tested the stability of edge weights by creating 5000 samples using non-parametric bootstrap (resampling rows with replacement). Case-drop bootstrapping was used to test how much percent of our original sample could be dropped to maintain an average correlation of r > .7 of the centrality parameter between the original and bootstrapped samples [44].

We conducted all of our analyses using the statistical software *JASP* [45] and *R* [46].

## Results

### Sample characteristics

As previously mentioned, we included a group factor in our manipulation checks and main analyses to control for potential differences between participants who reported a current or previous mental health issue. Therefore, we report descriptive statistics for both groups and the entire sample (see Table 1). Overall, our participants were rather young (*M* = 33.25, *SD* = 11.91), well-educated (83.87% with a high school diploma), and of varying wealth (*M =* 3308.78, *SD* = 5034.01). Additionally, 62.58% reported that they either currently suffer or have previously suffered from depression. On a descriptive level, the frequencies and means for all clinical variables differed as expected when comparing participants who reported no current or previous mental health issue with those who did (see Table 1).

**Table 1:** Sample characteristics.

	No Mental Health Issue (*N* = 81)	Mental Health Issue (*N* = 74)	All (*N* = 155)
**Gender**			
male, *N* (%)	45 (55.56)	27 (36.49)	72 (46.45)
female, *N* (%)	36 (44.44)	45 (60.81)	81 (52.26)
diverse, *N* (%)	0 (0.00)	2 (2.70)	2 (1.29)
**Citizenship**			
German, *N* (%)	80 (98.77)	70 (94.59)	150 (96.77)
mixed, *N* (%)	0 (0.00)	2 (2.70)	2 (1.29)
other, *N* (%)	1 (1.23)	2 (2.70)	3 (1.94)
**Legal status**			
unmarried, *N* (%)	62 (80.25)	62 (83.78)	124 (80.00)
married, *N* (%)	15 (18.52)	7 (9.46)	22 (14.19)
divorced, *N* (%)	2 (2.47)	3 (4.05)	5 (3.23)
widowed, *N* (%)	1 (1.23)	1 (1.35)	2 (1.29)
registered partnership, *N* (%)	1 (1.23)	1 (1.35)	2 (1.29)
**Relationship status**			
single, *N* (%)	37 (45.68)	34 (45.95)	77 (49.68)
partnership, *N* (%)	44 (54.32)	40 (54.05)	78 (50.32)
**Education**			
major school diploma, *N* (%)	1 (1.23)	1 (1.35)	2 (1.29)
secondary school diploma, *N* (%)	11 (13.58)	12 (16.22)	23 (14.84)
high school diploma, *N* (%)	69 (85.19)	61 (82.43)	130 (83.87)
**Vocation**			
none/not completed, *N* (%)	15 (18.52)	26 (35.14)	41 (26.45)
completed, *N* (%)	22 (27.16)	16 (21.62)	38 (24.52)
Bachelor’s degree, *N* (%)	23 (28.40)	15 (20.27)	38 (24.52)
Master’s degree, *N* (%)	20 (24.69)	15 (20.27)	35 (22.58)
PhD, *N* (%)	1 (1.23)	2 (2.70)	3 (1.94)
**Employment**			
full-time, *N* (%)	41 (50.62)	29 (39.19)	70 (45.16)
part-time, *N* (%)	24 (29.63)	24 (32.43)	48 (30.97)
not employed, *N* (%)	16 (19.75)	21 (28.38)	37 (23.87)
**Migration**			
immigrant, *N* (%)	5 (6.17)	3 (4.05)	8 (5.16)
no immigrant, *N* (%)	76 (93.83)	71 (95.95)	147 (94.84)
**Diagnosis of MDD**			
never, *N* (%)	81 (100.00)	16 (21.62)	97 (62.58)
currently or in the past, *N* (%)	0 (0.00)	58 (78.38)	58 (37.42)
**Outpatient treatment**			
never, *N* (%)	81 (100.00)	24 (32.43)	105 (67.74)
currently or in the past, *N* (%)	0 (0.00)	50 (67.57)	50 (32.26)
**Inpatient treatment**			
never, *N* (%)	81 (100.00)	50 (67.57)	131 (84.52)
currently or in the past, *N* (%)	0 (0.00)	24 (32.43)	24 (15.48)
**Medication**			
never, *N* (%)	79 (97.53)	41 (55.41)	120 (77.42)
currently or in the past, *N* (%)	2 (2.47)	33 (44.59)	35 (22.58)
**Age**, *M (SD)*	34.58 (12.59)	31.80 (11.02)	33.25 (11.91)
**Weekly working time**, *M (SD)*	25.25 (16.72)^b^	20.82 (16.99)^c^	23.14 (16.94)
**Household members**, *M (SD)*	2.56 (1.41)	2.10 (1.14)	2.34 (1.31)
**Net household income**, *M (SD)*	3018.50 (1845.71)^b^	3622.60 (7016.67)	3308.78 (5034.01)
**Disorder onset**	-	17.32 (8.65)	-
**BSI-18** ^**a**^			
overall	6.96 (7.90)	15.28 (10.59)	10.94 (10.14)
depression, *M (SD)*	2.85 (3.69)	6.80 (5.26)	4.74 (4.91)
anxiety, *M (SD)*	2.74 (2.78)	5.53 (4.27)	4.07 (3.82)
somatization, *M (SD)*	1.37 (2.41)	2.96 (3.16)	2.13 (2.89)
**DES** ^**a**^			
overall, *M (SD)*	51.30 (12.27)	60.96 (15.85)	55.91 (14.86)
social rejection, *M (SD)*	10.30 (3.39)	12.26 (4.92)	11.23 (4.29)
social support, *M (SD)*	6.82 (2.39)	7.49 (2.91)	7.14 (2.67)
mood regulation, *M (SD)*	12.48 (4.05)	15.14 (4.65)	13.75 (4.53)
ability to perform, *M (SD)*	4.46 (2.01)	5.76 (2.43)	5.08 (2.31)
**ASF-E** ^**a**^			
negative			
overall, *M (SD)*	106.89 (27.09)	118.45 (30.35)	112.44 (29.19)
internality, *M (SD)*	39.19 (9.40)	42.35 (9.45)	40.70 (9.53)
stability, *M (SD)*	36.51 (9.58)	39.68 (11.85)	38.02 (10.80)
globalism, *M (SD)*	31.10 (11.78)	36.42 (13.01)	33.64 (12.63)
positive			
overall, *M (SD)*	81.22 (13.40)	77.01 (14.65)	79.21 (14.12)
internality, *M (SD)*	25.41 (5.24)	24.28 (5.82)	24.87 (5.54)
stability, *M (SD)*	28.21 (4.35)	27.03 (4.78)	27.65 (4.58)
globalism, *M (SD)*	27.61 (6.64)	25.70 (7.59)	26.70 (7.15)
**PTQ-15**^**a**^, *M (SD)*	24.49 (14.12)	35.31 (15.14)	29.66 (15.54)
**SIAS-6**^**a**^, *M (SD)*	7.89 (5.40)	10.58 (5.84)	9.17 (5.76)
**SPS-6**^**a**^, *M (SD)*	4.96 (5.45)	8.99 (7.03)	6.88 (6.55)
**Surprise**			
after negative feedback, *M (SD)*	2.72 (1.11)	2.37 (1.14)	2.56 (1.14)
after positive feedback, *M (SD)*	1.62 (1.14)	2.20 (1.22)	1.90 (1.21)
**Feedback acceptance**			
after negative feedback, *M (SD)*	2.91 (1.02)	2.92 (0.99)	2.91 (1.00)
after positive feedback, *M (SD)*	3.84 (1.02)	3.50 (1.02)	3.68 (1.03)
**Immunizing cognition**			
after negative feedback, *M (SD)*	1.27 (0.86)	1.31 (0.78)	1.29 (0.82)
after positive feedback, *M (SD)*	1.38 (1.07)	1.46 (0.88)	1.42 (0.98)
**Distress due to participation**^**a**^,			
*M (SD)*	0.83 (0.95)	1.28 (1.09)	1.04 (1.04)

*N* = Sample size, *M =* Mean, *SD* = Standard deviation, MDD = Major depressive disorder, BSI-18 = 18-item version of the Brief Symptom Inventory, DES = Depressive Expectations Scale, ASF-E = Attributional Style Questionnaire for Adults, PTQ-15 = 15-item version of the Perseverative Thinking Questionnaire, SIAS-6 = 6-item version of the Social Anxiety Scale, SPS-6 = 6-item version of the Social Phobia Scale.

^a^ For reasons of comparability, we report mean sum scores here although we used mean composite scores in our main analyses.

^b^ These values are based on *n* = 80 participants.

^c^ This value is based on *n* = 73 participants.

### Manipulation checks

**Fig 3:**
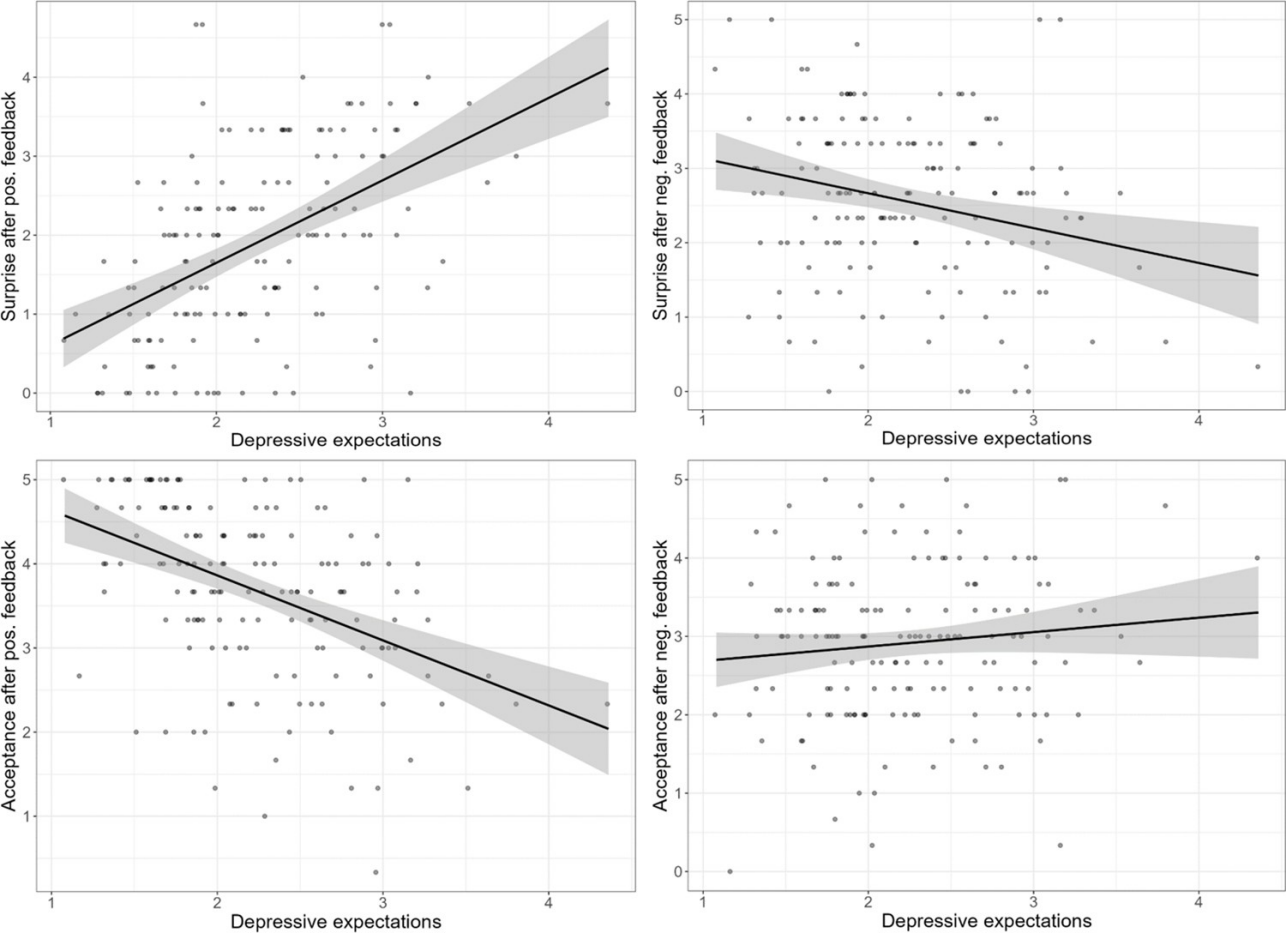
Effects of depressive cognition on surprise and feedback acceptance after social feedback. Left: After positive social feedback. Right: After negative social feedback.

Robust multiple linear regression analyses showed that, when controlling for all other predictor variables, *Depressive Expectations* had a significant positive effect on *Surprise after Positive Social Feedback* (*β =* 1.17, CI_95_ [0.80; 1.47]. *p* < .001; *F* (3, 151) = 19.679, *p* < .001, *R*^2^_adj._ = .27), and a significant negative effect on *Surprise after Negative Social Feedback* (*β =* -0.45, CI_95_ [-0.87; -0.06]. *p* = .021; *F* (3, 151) = 3.62, *p* = .015, *R*^2^_adj._ = .05). Moreover, it had a significant negative effect on *Feedback Acceptance after Positive Social Feedback* (*β =* -0.86, CI_95_ [-1.22; -0.53]. *p* < .001; *F* (3, 151) = 12.912, *p* < .001, *R*^2^_adj._ = .19) and no significant effect on *Feedback Acceptance after Negative Social Feedback* (*F* (3, 151) = 0.795, *p* = .498, *R*^2^_adj._ = 0). There were no further significant predictors. Taken together, these findings indicate that our manipulation was successful (Fig 3).

### Main analyses

**Fig 4:**
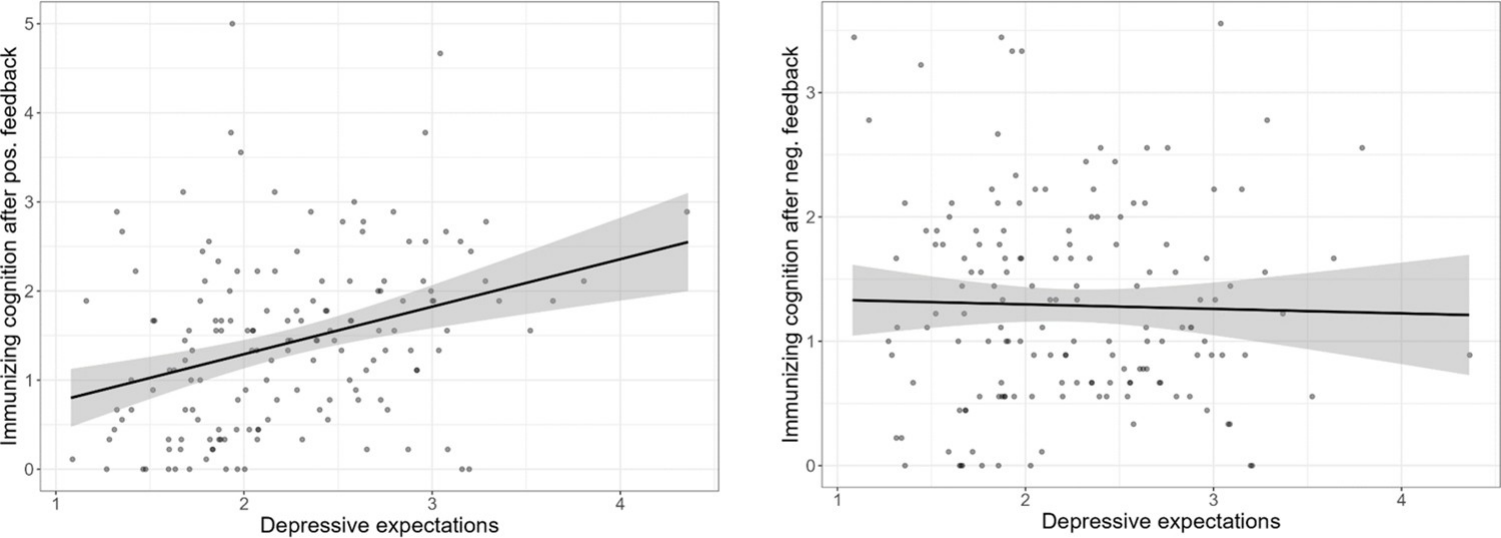
Effects of depressive cognition on immunizing cognition. Left: After positive social feedback. Right: After negative social feedback. Regression lines with 95% confidence band.

In line with H_1_, but contrary to H_2_, controlling for the influences of the other predictor variables, *Depressive Expectations* predicted higher levels of *Immunizing Cognition* (*β =* 0.51, CI_95_ [0.20; 0.80]. *p* = .002; *F* (3, 151) = 6.328, *p* < .001, *R*^2^_adj._ = .09) in response to positive social feedback, but not in response to negative social feedback (*F* (3, 151) = 1.647, *p* = .181, *R*^2^_adj._ = .01) (Fig 4).

When we included *Social Anxiety* and *Social Phobia* in the regression term, the effect of *Depressive Expectations* on *Immunizing Cognition* was attenuated (*β =* 0.35, CI_95_ [-0.02; 0.73]. *p* = .066; *F* (5, 149) = 4.385, *p* < .001, *R*^2^_adj._ = .10). Changes in *R*^2^ were insubstantial in this case (Δ *R*^2^ = 0.2, *F* (2, 149) = 1.419, *p* = .245). There were no significant predictors of *Immunizing Cognition* in response to negative social feedback (*ps >* .005).

Overall, these results are in line with H_1_ but inconsistent with H_2_.

### Exploratory network analysis

**Fig 5:**
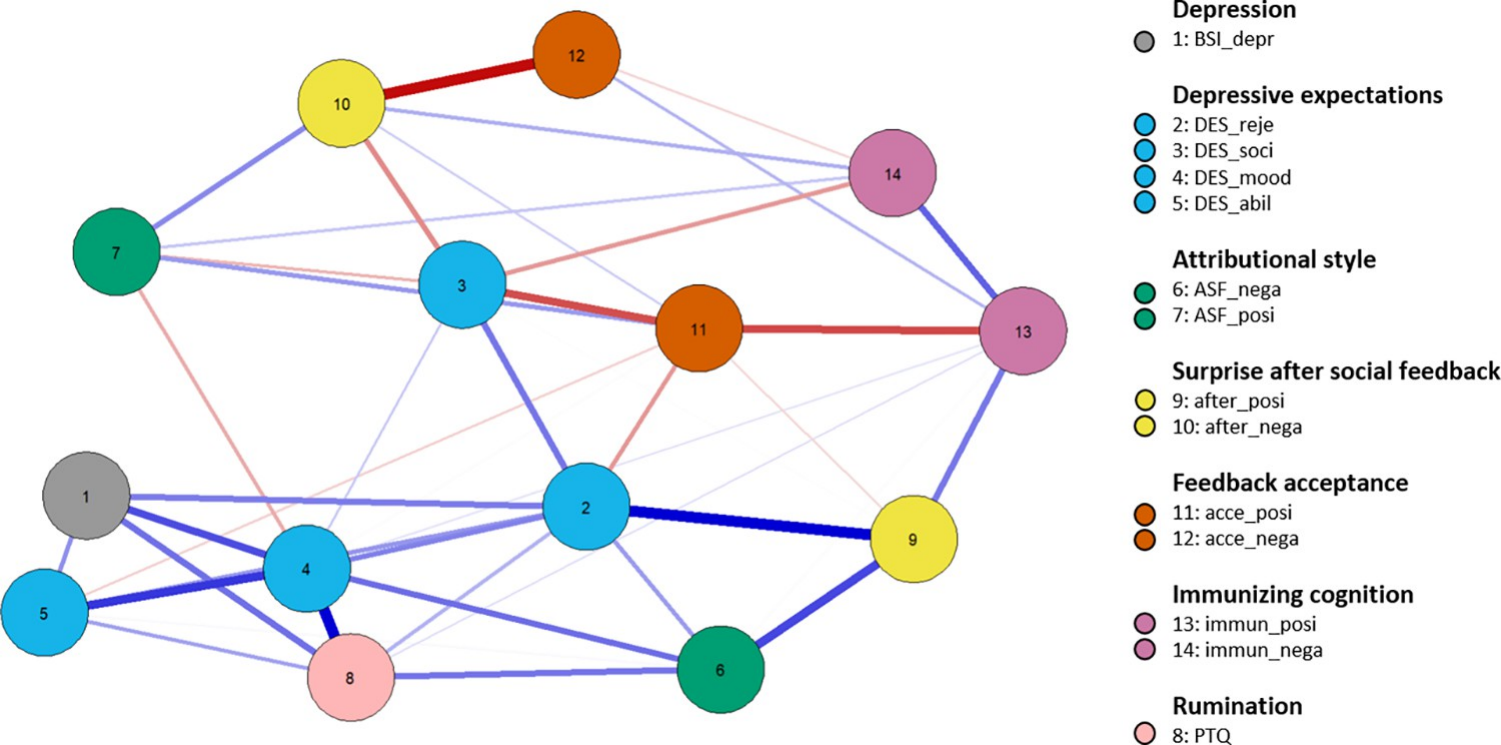
Exploratory network analysis. Blue indicates positive and red indicates negative edge weights. BSI_depr = “depression” subscale of the BSI-18, DES_reje = “rejection” subscale of the DES, DES_soci = “social support” subscale of the DES, DES_mood = “mood regulation” subscale of the DES, DES_abil = “ability to perform” subscale of the DES, ASF_nega = “negative attributional style” composite of the ASF, ASF_posi = “positive attributional style” composite of the ASF, after_pos = “surprise after positive social feedback” composite, after_nega = “surprise after negative social feedback” composite, accep_posi = “acceptance after positive social feedback” composite, accep_nega = “acceptance after negative social feedback” composite, immu_nega = “immunizing cognition after negative social feedback” composite, immun_posi = “immunizing cognition after positive social feedback” composite.

**Fig 6:**
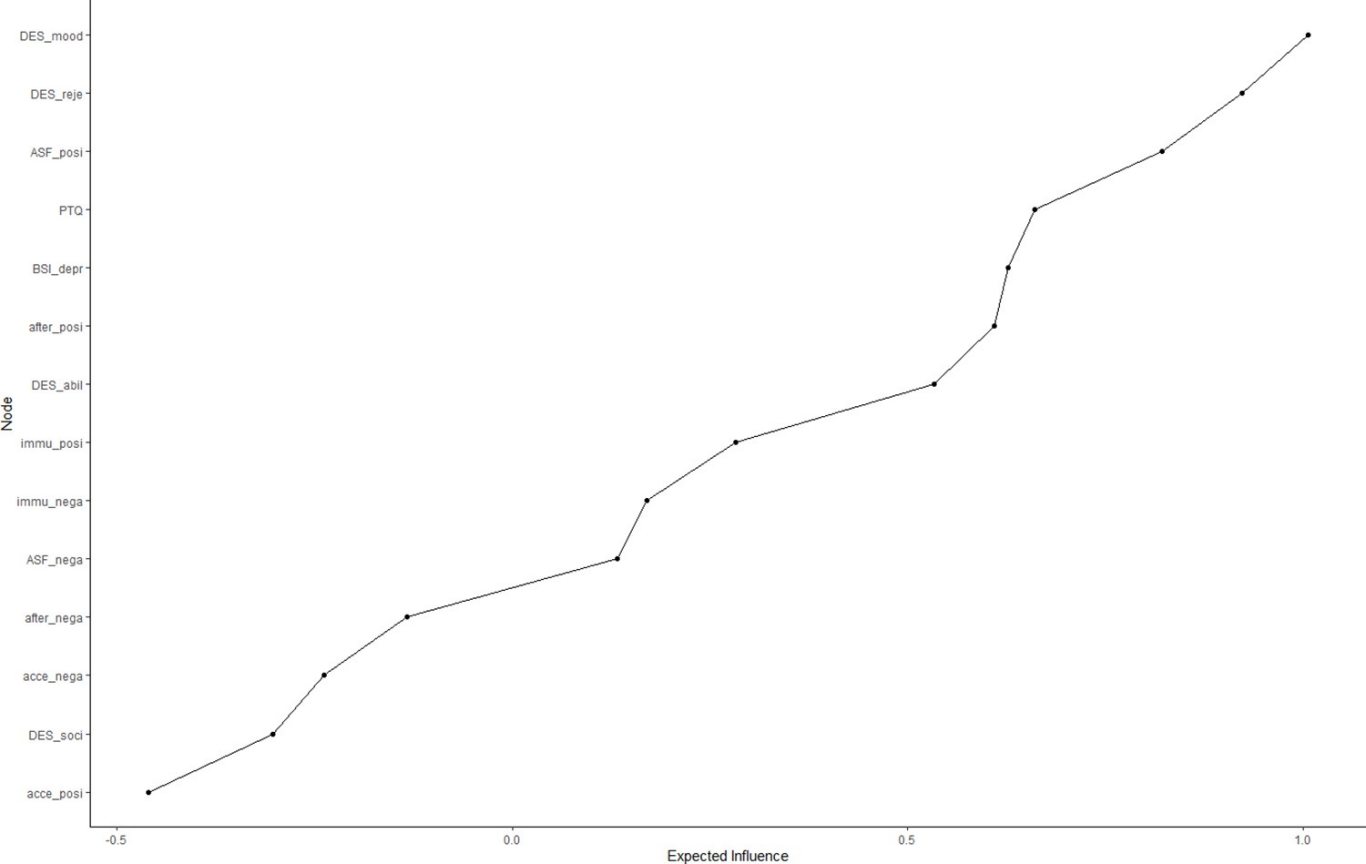
Expected influence of individual nodes. This graph illustrates the expected influence of each node in the network.

Fig 5 illustrates the exploratory network analysis of cognitive processing of unexpected social feedback. It describes the interplay and relative importance of *Depression*, *Depressive Expectations*, *Surprise*, *Feedback Acceptance after Social Feedback*, *Immunizing Cognition*, *Attributional Style*, and *Perseverative Negative Thinking*. The network consisted of 14 nodes with 42 (46.15%) of 91 possible edges. The expected influence for each node is found in Fig 6, along with the stability of each node’s respective connections in S4 Appendix. S5 Appendix demonstrates that we can remove approximately 28.4% of our sample without reducing the mean correlation between the expected influence from the original sample and expected influence from the 5000 bootstraps below .7. This indicates sufficient stability of the network analysis, and suggests that our results can be generalized.

Self-efficacy expectations for handling negative feelings had the strongest positive expected influence on the network, while acceptance of positive social feedback had the strongest negative expected influence. *Immunizing Cognition* was only indirectly related to attributional style and rumination processes, as were depressive symptoms. Interestingly, individuals showing higher levels of immunizing cognition after negative social feedback also appeared to immunize more strongly after positive experiences. However, this was associated with lower feedback acceptance only in the case of positive social feedback.

## Discussion

The aim of this study was to investigate the relationship between levels of depression, immunizing cognition, and related constructs in response to surprisingly positive versus negative social feedback.

### Hypotheses 1 and 2

In support of H_1_, we found that cognitive indicators of depression (i.e., depressive expectations) were associated with higher levels of immunizing cognition (as well as higher levels of surprise and lower levels of feedback acceptance) in response to unexpected social feedback, but only when processing surprisingly positive social feedback. However, in the case of immunizing cognition, this result was attenuated after controlling for social anxiety and symptoms of social phobia.

Although depression levels were associated with lower surprise, they were not associated with higher levels of feedback acceptance or lower levels of immunizing cognition after negative social feedback which is in contrast to H_2_. This suggests that depression levels were not associated with altered belief-updating following negative social feedback.

Taken together, these findings are consistent with previous research on belief-updating, suggesting that individuals with depressive symptoms have difficulty using “good news” to update prior beliefs compared to healthy controls [47–51]. This interpretation is consistent with reports on dispositional optimism, which suggests that healthy individuals tend to update their beliefs in an optimistically biased manner [51–54], whereas depressed individuals do not exhibit this bias [48, 50, 55, 56]. However, note that there is evidence challenging the idea of an optimism bias in healthy individuals (for critique and contradictory evidence, see [57–61]; for replies, see [62–65]).

Our findings are also consistent with reports in the literature on “depressive realism” suggesting that individuals with depressive symptoms have “more accurate” (i.e., less) belief-updating after positive feedback than healthy controls [66]. However, findings related to depressive realism have been mixed (e.g., [67]).

Moreover, our results on H_1_ align with the evidence suggesting that individuals with depressive symptoms exhibit blunted reward sensitivity to positive stimuli (for a synthesis, see [68]) and a lack of positive interpretation biases [69]. However, our results on H_2_ raise questions, as evidence suggests dysfunctional punishment and negative information processing in depression (e.g., [69, 70]; for syntheses, see [71–75]). We suspect that the contradiction between our results regarding H_2_ and the literature is due to the sheer complexity of belief updating and cognitive immunization processes. These processes involve sub-processes and mediating variables that–depending on the feedback’s valence—may interact differently with depressive symptoms and belief-updating (see our exploratory findings).

### Exploratory findings

In addition to the points discussed above, our network analysis revealed insights regarding the interplay of social feedback, surprise, immunizing cognition, feedback acceptance, and other variables. Specifically, higher surprise levels were directly linked to less feedback acceptance and higher levels of immunizing cognition, regardless of the feedback’s valence. However, the link between surprise level and positive feedback acceptance was rather weak in our estimation. These results highlight the importance of developing interventions that inhibit cognitive immunization in clinical practice [19, 23]. Moreover, they are consistent with evidence suggesting that moderate, not maximum disconfirmation of expectations is effective in modifying dysfunctional beliefs [26, 76, 77].

Moreover, our exploratory analyses suggested weak and mostly indirect connections between immunizing cognition and attributional style or rumination. This is somewhat surprising considering prior theorizing [23, 24]. It suggests that immunization should be further investigated in the context of motivational factors, such as “costs for change” [23] or subjective “utility” [78].

Interestingly, regarding our expected influence estimates, we conclude that working on accepting positive social feedback (node 11) and building self-efficacy in dealing with negative feelings (node 4) constitute fruitful targets for developing treatments. This aligns with research that highlights the role of impaired positive information processing (e.g., [79]; for syntheses, see [72, 80]) and mood regulation deficits in depression (e.g., [81]; for syntheses, see [73–75, 82, 83]). Clinicians can promote the acceptance of positive social feedback through various strategies. For instance, research on biased belief updating in depression suggests that practitioners should take the following steps: (a) reduce the negative mood of depressed individuals before presenting them with unexpectedly positive social feedback, (b) provide feedback that is positive yet still credible from the patient’s perspective, and (c) inform patients about immunizing thoughts beforehand to prevent cognitive immunization [19]. Other authors also recommend encouraging patients to verbalize their expectations and perceptions before and during encounters with surprising experiences, and to direct patients’ attention to stimuli that challenge their expectations [23]. Finally, Craske and colleagues [84, 85] suggest varying the conditions and behaviors in which patients encounter expectation-violating experiences. In terms of promoting self-efficacy in dealing with negative emotions, various strategies have also been suggested, such as focusing on savoring training or aiming to expand the repertoire of emotion regulation strategies [82].

### Limitations

Our study has limitations that we should point out. Despite its strengths, such as applying naturalistic stimuli and innovative measurement of immunizing cognition, the following points should be noted.

First, our data were collected online, which did not allow for in-depth structured clinical diagnostics. While this enabled us to recruit a heterogeneous sample with sufficient variability in the variables investigated, it may have limited our study’s internal validity and the generalizability of our findings to clinical samples. Future research should aim to replicate our findings in more controlled settings.

Secondly, the social feedback presented to participants did not come from real social relationships but from strangers, which reduces the comparability with authentic real-life social feedback and calls into question the validity of the manipulation with respect to cognitive immunization and belief-updating. However, this interpretation is somewhat countered by our results from the pretests and manipulation checks that suggest the validity of the stimuli. Nonetheless, future research should aim to replicate our findings in real-life social settings and explore the effects on belief-updating within patients’ real social environments.

Thirdly, there was only moderate agreement between raters regarding participants’ thoughts after social feedback. This might imply potential weaknesses in either the construct’s definition or its operationalization. Alternatively, the subclinical nature of the sample could have restricted variance. Future studies should explore whether inter-rater agreement and associations with depression increase when clinical samples are tested.

Fourthly, our network analysis results should be interpreted with caution, as not all potentially relevant variables could be included in our model. Moreover, this analysis does not allow for causal interpretation, as it was based on a Gaussian Graphical Model with cross-sectional data. Moreover, note that the connections’ stability between individual nodes requires careful interpretation, as our sample size was rather modest.

Finally, one could argue that we should have relied on self-report measures of cognitive immunization (as in previous studies). However, we believe that capturing cognitive immunization only via self-reports has considerable drawbacks, especially knowing that cognitive immunization is a (partially) implicit phenomenon. Future studies should therefore focus on adding implicit measures to capture different facets of cognitive immunization. Additionally, when measuring cognitive immunization, we suggest distinguishing the inclination to generate immunizing cognitions from the resulting outcomes, such as less belief-updating and decreased feedback-acceptance.

## Conclusion

In conclusion, we investigated in this study the relationship between depressive symptoms, feedback acceptance, immunizing cognition, and related constructs in the context of both surprisingly positive and negative social feedback. Our main analyses revealed that cognitive indicators of depression were associated with higher levels of surprise, less feedback acceptance, and higher levels of immunizing cognition in response to surprisingly positive social feedback. However, depression levels were not associated with higher levels of feedback acceptance or lower levels of immunizing cognition after negative social feedback. These findings extend previous research to the social domain, suggesting that individuals with depressive symptoms have problems in using “good news” to update prior beliefs.

Regarding the interaction with attributional style or rumination processes, we observed weak and mostly indirect associations with cognitive immunization. This may fit well with the idea of a discrete construct that is more closely connected to motivational factors.

In terms of clinical practice, our analyses underscore the importance of promoting acceptance and inhibiting immunizing cognitions in the context of positive social feedback. It also suggests promoting moderate rather than maximum disconfirmation when aiming to update dysfunctional social beliefs.

## Supporting information

S1 Appendix Criteria for rating participants’ thoughts. (DOCX)

S2 Appendix English translations of the initial pool of feedback statements by valence. (DOCX)

S3 Appendix Pilot test of the study procedure and decision on using audio or video stimuli in the main study. (DOCX)

S4 Appendix Stability of edge weights. The red graph corresponds to the edge estimate in the sample, the black graph to the mean edge estimate in the bootstrapped samples and the gray area to the 95% confidence interval band from the bootstraps edge weights.
(PPTX)

S5 Appendix Mean correlation between expected influence in the sample compared to the 5000 bootstrapped samples for each exclusion interval. (PPTX)

## References

[1] Duque A, Vázquez C. Double attention bias for positive and negative emotional faces in clinical depression: evidence from an eye-tracking study. J Behav Ther Exp Psychiatry [Internet]. 2015 Mar;46:107–14. Available from: https://linkinghub.elsevier.com/retrieve/pii/S0005791614000810 doi: 10.1016/j.jbtep.2014.09.005 25305417

[2] Smith HL, Summers BJ, Dillon KH, Macatee RJ, Cougle JR. Hostile interpretation bias in depression. J Affect Disord [Internet]. 2016 Oct;203:9–13. Available from: doi: 10.1016/j.jad.2016.05.070 27267952

[3] Sheets ES, Armey MF. Daily interpersonal and noninterpersonal stress reactivity in current and remitted depression. Cognit Ther Res [Internet]. 2020 Aug 23;44(4):774–87. Available from: 10.1007/s10608-020-10096-2

[4] Harkness KL, Stewart JG, Wynne-Edwards KE. Cortisol reactivity to social stress in adolescents: role of depression severity and child maltreatment. Psychoneuroendocrinology [Internet]. 2011 Feb;36(2):173–81. Available from: https://www.sciencedirect.com/science/article/pii/S0306453010001745 doi: 10.1016/j.psyneuen.2010.07.006 20688438

[5] Fernández-Theoduloz G, Paz V, Nicolaisen-Sobesky E, Pérez A, Buunk AP, Cabana Á, et al. Social avoidance in depression: a study using a social decision-making task. J Abnorm Psychol [Internet]. 2019 Apr 1 [cited 2022 May 23];128(3):234–44. Available from: https://pubmed.ncbi.nlm.nih.gov/30920233/ doi: 10.1037/abn0000415 30920233

[6] Levens SM, Gotlib IH. Updating positive and negative stimuli in working memory in depression. J Exp Psychol Gen [Internet]. 2010;139(4):654–64. Available from: www.apa.org/pubs/journals/XGE doi: 10.1037/a0020283 21038984 PMC2984552

[7] Wang Y, Zhou Y, Li S, Wang P, Wu GW, Liu ZN. Impaired social decision making in patients with major depressive disorder. BMC Psychiatry [Internet]. 2014 Dec 23;14(1):18. Available from: http://www.biomedcentral.com/1471-244X/14/18 doi: 10.1186/1471-244X-14-18 24450703 PMC3904421

[8] Joormann J, Gotlib IH. Is this happiness I see?—Biases in the identification of emotional facial expressions in depression and social phobia. J Abnorm Psychol [Internet]. 2006 Nov;115(4):705–14. Available from: http://doi.apa.org/getdoi.cfm?doi=10.1037/0021-843X.115.4.705 17100528 10.1037/0021-843X.115.4.705

[9] Thoma P, Schmidt T, Juckel G, Norra C, Suchan B. Nice or effective?—Social problem solving strategies in patients with major depressive disorder. Psychiatry Res [Internet]. 2015 Aug;228(3):835–42. Available from: doi: 10.1016/j.psychres.2015.05.015 26051176

[10] Hames JL, Hagan CR, Joiner TE. Interpersonal processes in depression. Annu Rev Clin Psychol [Internet]. 2013 Mar 28;9(1):355–77. Available from: doi: 10.1146/annurev-clinpsy-050212-185553 23297787

[11] Kupferberg A, Hasler G. The social cost of depression: investigating the impact of impaired social emotion regulation, social cognition, and interpersonal behavior on social functioning. J Affect Disord Reports [Internet]. 2023 Dec;14(December 2022):100631. Available from: 10.1016/j.jadr.2023.100631

[12] Segrin C. Social skills deficits associated with depression. Clin Psychol Rev [Internet]. 2000 Apr [cited 2022 May 23];20(3):379–403. Available from: https://pubmed.ncbi.nlm.nih.gov/10779900/ doi: 10.1016/s0272-7358(98)00104-4 10779900

[13] Santini ZI, Koyanagi A, Tyrovolas S, Mason C, Haro JM. The association between social relationships and depression: a systematic review. J Affect Disord [Internet]. 2015 Apr;175:53–65. Available from: doi: 10.1016/j.jad.2014.12.049 25594512

[14] Badcock PB, Davey CG, Whittle S, Allen NB, Friston KJ. The depressed brain: an evolutionary systems theory. Trends Cogn Sci [Internet]. 2017 Mar 1 [cited 2022 May 23];21(3):182–94. Available from: https://linkinghub.elsevier.com/retrieve/pii/S1364661317300050 doi: 10.1016/j.tics.2017.01.005 28161288

[15] Nanni V, Uher R, Danese A. Childhood maltreatment predicts unfavorable course of illness and treatment outcome in depression: a meta-analysis. Am J Psychiatry [Internet]. 2012 Feb [cited 2022 May 23];169(2):141–51. Available from: https://pubmed.ncbi.nlm.nih.gov/22420036/ doi: 10.1176/appi.ajp.2011.11020335 22420036

[16] Bishop A, Younan R, Low J, Pilkington PD. Early maladaptive schemas and depression in adulthood: a systematic review and meta‐analysis. Clin Psychol Psychother [Internet]. 2022 Jan 16 [cited 2022 May 23];29(1):111–30. Available from: https://onlinelibrary.wiley.com/doi/full/10.1002/cpp.2630 34131990 10.1002/cpp.2630

[17] Rief W, Joormann J. Revisiting the cognitive model of depression: the role of expectations. Clin Psychol Eur [Internet]. 2019 Mar 29 [cited 2022 May 23];1(1):1–19. Available from: https://cpe.psychopen.eu/index.php/cpe/article/view/2373/2373.html

[18] Kube T, Schwarting R, Rozenkrantz L, Glombiewski JA, Rief W. Distorted cognitive processes in major depression: a predictive processing perspective. Biol Psychiatry [Internet]. 2020 Mar;87(5):388–98. Available from: https://linkinghub.elsevier.com/retrieve/pii/S0006322319315501 doi: 10.1016/j.biopsych.2019.07.017 31515055

[19] Kube T. Biased belief updating in depression. Clin Psychol Rev [Internet]. 2023 Jun;103(October 2022):102298. Available from: doi: 10.1016/j.cpr.2023.102298 37290245

[20] Liu RT, Alloy LB. Stress generation in depression: a systematic review of the empirical literature and recommendations for future study. Clin Psychol Rev [Internet]. 2010 Jul;30(5):582–93. Available from: doi: 10.1016/j.cpr.2010.04.010 20478648 PMC3049314

[21] Kirchner L, Schummer SE, Krug H, Kube T, Rief W. How social rejection expectations and depressive symptoms bi‐directionally predict each other–a cross‐lagged panel analysis. Psychol Psychother Theory, Res Pract [Internet]. 2022 Jun [cited 2022 May 23];95(2):477–92. Available from: https://onlinelibrary.wiley.com/doi/10.1111/papt.12383 35099102 10.1111/papt.12383

[22] Beeson CML, Brittain H, Vaillancourt T. The temporal precedence of peer rejection, rejection sensitivity, depression, and aggression across adolescence. Child Psychiatry Hum Dev [Internet]. 2020 Oct 28 [cited 2022 May 23];51(5):781–91. Available from: https://pubmed.ncbi.nlm.nih.gov/32462359/ doi: 10.1007/s10578-020-01008-2 32462359

[23] Rief W, Sperl MFJ, Braun-Koch K, Khosrowtaj Z, Kirchner L, Schäfer L, et al. Using expectation violation models to improve the outcome of psychological treatments. Clin Psychol Rev. 2022;98(June). doi: 10.1016/j.cpr.2022.102212 36371900

[24] Panitz C, Endres D, Buchholz M, Khosrowtaj Z, Sperl MFJ, Mueller EM, et al. A revised framework for the investigation of expectation update versus maintenance in the context of expectation violations: the ViolEx 2.0 model. Front Psychol [Internet]. 2021 Nov 11;12(November). Available from: https://www.frontiersin.org/articles/10.3389/fpsyg.2021.726432/full 34858264 10.3389/fpsyg.2021.726432 PMC8632008

[25] Berg M, Feldmann M, Kirchner L, Kube T. Oversampled and undersolved: depressive rumination from an active inference perspective. Neurosci Biobehav Rev [Internet]. 2022 Nov;142(August):104873. Available from: https://linkinghub.elsevier.com/retrieve/pii/S0149763422003621 doi: 10.1016/j.neubiorev.2022.104873 36116573

[26] Kube T, Kirchner L, Lemmer G, Glombiewski JA. How the discrepancy between prior expectations and new information influences expectation updating in depression—the greater, the better? Clin Psychol Sci [Internet]. 2022 May 29;10(3):430–49. Available from: 10.1177/21677026211024644

[27] Kube T, Kirchner L, Rief W, Gärtner T, Glombiewski JA. Belief updating in depression is not related to increased sensitivity to unexpectedly negative information. Behav Res Ther [Internet]. 2019 Dec 1 [cited 2022 Aug 10];123(May):103509. Available from: 10.1016/j.brat.2019.103509 31715323

[28] Kube T, Rief W, Gollwitzer M, Gärtner T, Glombiewski JA. Why dysfunctional expectations in depression persist–results from two experimental studies investigating cognitive immunization. Psychol Med [Internet]. 2019 Jul 22;49(09):1532–44. Available from: https://www.cambridge.org/core/product/identifier/S0033291718002106/type/journal_article doi: 10.1017/S0033291718002106 30131084

[29] Kube T, Glombiewski JA. No evidence for the involvement of cognitive immunisation in updating beliefs about the self in three non-clinical samples. Cognit Ther Res [Internet]. 2022 Feb;46(1):43–61. Available from: 10.1007/s10608-021-10256-y 34345057 PMC8323093

[30] Kube T, Glombiewski JA, Gall J, Touissant L, Gärtner T, Rief W. How to modify persisting negative expectations in major depression?—An experimental study comparing three strategies to inhibit cognitive immunization against novel positive experiences. J Affect Disord [Internet]. 2019 May 1 [cited 2023 Mar 24];250(February):231–40. Available from: https://linkinghub.elsevier.com/retrieve/pii/S0165032718332737 doi: 10.1016/j.jad.2019.03.027 30870773

[31] Kube T. If the discrepancy between expectations and actual information is too large, expectation change decreases–a replication study. J Behav Ther Exp Psychiatry [Internet]. 2023 Jun 1 [cited 2023 Mar 24];79:101831. Available from: https://linkinghub.elsevier.com/retrieve/pii/S0005791622001094 doi: 10.1016/j.jbtep.2022.101831 36521199

[32] D’Astolfo L, Kirchner L, Rief W. No1LikesU!–A pilot study on an ecologically valid and highly standardised experimental paradigm to investigate social rejection expectations and their modification. Clin Psychol Eur [Internet]. 2020 Jun 30;2(2):1–21. Available from: https://cpe.psychopen.eu/index.php/cpe/article/view/2997 10.32872/cpe.v2i2.2997 PMC9645489 36397828

[33] Groth RM, Rief W. Response to unexpected social inclusion: A study using the cyberball paradigm. Front Psychiatry. 2022;13(Mdd). doi: 10.3389/fpsyt.2022.911950 35990056 PMC9381977

[34] Spitzer C, Kendzierski T, Volkmann J, Franke G. The short version of the Brief Symptom Inventory (BSI-18): preliminary psychometric properties of the German translation. PPmP—Psychother · Psychosom · Medizinische Psychol [Internet]. 2011 Feb;58(02):517–23. Available from: http://www.thieme-connect.de/DOI/DOI?10.1055/s-2008-1061525 10.1055/s-0031-1281602 21870312

[35] Dunn TJ, Baguley T, Brunsden V. From alpha to omega: a practical solution to the pervasive problem of internal consistency estimation. Br J Psychol [Internet]. 2014 Aug;105(3):399–412. Available from: https://onlinelibrary.wiley.com/doi/10.1111/bjop.12046 24844115 10.1111/bjop.12046

[36] Kube T D’Astolfo L, Glombiewski JA, Doering BK, Rief W Focusing on situation-specific expectations in major depression as basis for behavioural experiments–development of the Depressive Expectations Scale. Psychol Psychother Theory, Res Pract. 2017;90(3):336–52. doi: 10.1111/papt.12114 27935247

[37] Cohen J. A coefficient of agreement for nominal scales. Educ Psychol Meas [Internet]. 1960 Apr 2;20(1):37–46. Available from: http://journals.sagepub.com/doi/10.1177/001316446002000104

[38] Poppe P, Stiensmeier-Pelster J, Pelster A. Attributionsstilfragebogen für Erwachsene. Göttingen: Hogrefe; 2005.

[39] Ehring T, Zetsche U, Weidacker K, Wahl K, Schönfeld S, Ehlers A. The Perseverative Thinking Questionnaire (PTQ): validation of a content-independent measure of repetitive negative thinking. J Behav Ther Exp Psychiatry [Internet]. 2011 Jun;42(2):225–32. Available from: doi: 10.1016/j.jbtep.2010.12.003 21315886 PMC3042595

[40] Peters L, Sunderland M, Andrews G, Rapee RM, Mattick RP. Development of a short form Social Interaction Anxiety (SIAS) and Social Phobia Scale (SPS) using nonparametric item response theory: The SIAS-6 and the SPS-6. Psychol Assess [Internet]. 2012;24(1):66–76. Available from: http://doi.apa.org/getdoi.cfm?doi=10.1037/a0024544 21744971 10.1037/a0024544

[41] Friedman J, Hastie T, Tibshirani R. Sparse inverse covariance estimation with the graphical lasso. Biostatistics [Internet]. 2008 Jul 1 [cited 2023 Apr 13];9(3):432–41. Available from: https://academic.oup.com/biostatistics/article/9/3/432/224260 doi: 10.1093/biostatistics/kxm045 18079126 PMC3019769

[42] Foygel R, Drton M. Extended Bayesian information criteria for Gaussian graphical models. 2010 Nov 30; Available from: http://arxiv.org/abs/1011.6640

[43] Robinaugh DJ, Millner AJ, McNally RJ. Identifying highly influential nodes in the complicated grief network. J Abnorm Psychol [Internet]. 2016 Aug;125(6):747–57. Available from: http://doi.apa.org/getdoi.cfm?doi=10.1037/abn0000181 27505622 10.1037/abn0000181 PMC5060093

[44] Epskamp S, Fried EI. A tutorial on regularized partial correlation networks. Psychol Methods [Internet]. 2018 Dec;23(4):617–34. Available from: http://doi.apa.org/getdoi.cfm?doi=10.1037/met0000167 29595293 10.1037/met0000167

[45] Team JASP. JASP [Internet]. 2023. Available from: https://jasp-stats.org/

[46] R Core Team. R: A Language and environment for statistical computing. [Internet]. 2021. Available from: https://cran.r-project.org

[47] Kube T, Glombiewski JA. How depressive symptoms hinder positive information processing: an experimental study on the interplay of cognitive immunisation and negative mood in the context of expectation adjustment. Cognit Ther Res [Internet]. 2021 Jun 22;45(3):517–28. Available from: 10.1007/s10608-020-10191-4

[48] Korn CW, Sharot T, Walter H, Heekeren HR, Dolan RJ. Depression is related to an absence of optimistically biased belief updating about future life events. Psychol Med [Internet]. 2013 Feb 15;44(3):579–92. Available from: https://www.cambridge.org/core/product/identifier/S0033291713001074/type/journal_article doi: 10.1017/S0033291713001074 23672737 PMC3880066

[49] Conklin LR, Strunk DR, Fazio RH. Attitude formation in depression: evidence for deficits in forming positive attitudes. J Behav Ther Exp Psychiatry [Internet]. 2009 Mar;40(1):120–6. Available from: doi: 10.1016/j.jbtep.2008.07.001 18757051

[50] Hobbs C, Vozarova P, Sabharwal A, Shah P, Button K. Is depression associated with reduced optimistic belief updating? R Soc Open Sci. 2022;9(2). doi: 10.1098/rsos.190814 35127107 PMC8808098

[51] Sharot T. The optimism bias. Curr Biol [Internet]. 2011 Dec;21(23):R941–5. Available from: doi: 10.1016/j.cub.2011.10.030 22153158

[52] Kuzmanovic B, Rigoux L. Valence-dependent belief updating: computational validation. Front Psychol [Internet]. 2017 Jun 29;8:1087. Available from: www.frontiersin.org doi: 10.3389/fpsyg.2017.01087 28706499 PMC5489622

[53] Kuzmanovic B, Jefferson A, Vogeley K. The role of the neural reward circuitry in self-referential optimistic belief updates. Neuroimage [Internet]. 2016 Jun;133:151–62. Available from: doi: 10.1016/j.neuroimage.2016.02.014 26883063

[54] Kuzmanovic B, Jefferson A, Vogeley K. Self-specific optimism bias in belief updating is associated with high trait optimism. J Behav Decis Mak [Internet]. 2015 Jul;28(3):281–93. Available from: https://onlinelibrary.wiley.com/doi/ doi: 10.1002/bdm.1849

[55] Strunk DR, Adler AD. Cognitive biases in three prediction tasks: a test of the cognitive model of depression. Behav Res Ther [Internet]. 2009 Jan;47(1):34–40. Available from: doi: 10.1016/j.brat.2008.10.008 19010460

[56] Strunk DR, Lopez H, DeRubeis RJ. Depressive symptoms are associated with unrealistic negative predictions of future life events. Behav Res Ther [Internet]. 2006 Jun 1 [cited 2023 Mar 28];44(6):861–82. Available from: https://linkinghub.elsevier.com/retrieve/pii/S0005796705001476 doi: 10.1016/j.brat.2005.07.001 16126162

[57] Burton JW, Harris AJL, Shah P, Hahn U. Optimism where there is none: asymmetric belief updating observed with valence-neutral life events. Cognition [Internet]. 2022 Jan 1 [cited 2023 Mar 28];218:104939. Available from: https://linkinghub.elsevier.com/retrieve/pii/S0010027721003620 doi: 10.1016/j.cognition.2021.104939 34717257

[58] Powell D. A descriptive Bayesian account of optimism in belief revision. In: Proceedings of the Annual Meeting of the Cognitive Science Society. 2022.

[59] Harris AJL, Hahn U. Unrealistic optimism about future life events: a cautionary note. Psychol Rev [Internet]. 2011 Jan;118(1):135–54. Available from: http://doi.apa.org/getdoi.cfm?doi=10.1037/a0020997 21058872 10.1037/a0020997

[60] Harris AJL, de Molière L, Soh M, Hahn U. Unrealistic comparative optimism: an unsuccessful search for evidence of a genuinely motivational bias. Yechiam E, editor. PLoS One [Internet]. 2017 Mar 9;12(3):e0173136. Available from: https://dx.plos.org/10.1371/journal.pone.0173136 28278200 10.1371/journal.pone.0173136 PMC5344342

[61] Shah P, Harris AJL, Bird G, Catmur C, Hahn U. A pessimistic view of optimistic belief updating. Cogn Psychol [Internet]. 2016 Nov;90:71–127. Available from: doi: 10.1016/j.cogpsych.2016.05.004 27542765

[62] Marks J, Baines S. Optimistic belief updating despite inclusion of positive events. Learn Motiv [Internet]. 2017 May;58(June):88–101. Available from: 10.1016/j.lmot.2017.05.001

[63] Garrett N, Sharot T. Optimistic update bias holds firm: three tests of robustness following Shah et al. Conscious Cogn [Internet]. 2017 Apr 1 [cited 2023 Mar 28];50:12–22. Available from: https://linkinghub.elsevier.com/retrieve/pii/S1053810016300800 doi: 10.1016/j.concog.2016.10.013 27836628 PMC5380127

[64] Sharot T, Garrett N. A guideline and cautionary note: how to use the belief update task correctly. Methods Psychol [Internet]. 2022 Nov;6(April):100091. Available from: https://linkinghub.elsevier.com/retrieve/pii/S2590260122000029

[65] Garrett N, Sharot T. Failure to replicate Burton, Harris, Shah & Hahn (2021): there is no belief update bias for neutral events. 10.1080/20445911.2023.2245112 PMC10591604 38013976

[66] Moore MT, Fresco DM. Depressive realism: a meta-analytic review. Clin Psychol Rev [Internet]. 2012 Aug;32(6):496–509. Available from: doi: 10.1016/j.cpr.2012.05.004 22717337

[67] Ackermann R, DeRubeis RJ. Is depressive realism real? Clin Psychol Rev. 1991;11(5):565–84.

[68] Alloy LB, Olino T, Freed RD, Nusslock R. Role of reward sensitivity and processing in major depressive and bipolar spectrum disorders. Behav Ther [Internet]. 2016 Sep;47(5):600–21. Available from: doi: 10.1016/j.beth.2016.02.014 27816074 PMC5119651

[69] Everaert J, Bronstein M V., Cannon TD, Joormann J. Looking through tinted glasses: depression and social anxiety are related to both interpretation biases and inflexible negative interpretations. Clin Psychol Sci. 2018 Jul;6(4):517–28.

[70] Garrett N, Sharot T, Faulkner P, Korn CW, Roiser JP, Dolan RJ. Losing the rose tinted glasses: neural substrates of unbiased belief updating in depression. Front Hum Neurosci [Internet]. 2014 Aug 28;8. Available from: www.frontiersin.org doi: 10.3389/fnhum.2014.00639 25221492 PMC4147849

[71] Everaert J, Podina IR, Koster EHW. A comprehensive meta-analysis of interpretation biases in depression. Clin Psychol Rev [Internet]. 2017 Dec 1 [cited 2022 May 23];58:33–48. Available from: https://pubmed.ncbi.nlm.nih.gov/28974339/ doi: 10.1016/j.cpr.2017.09.005 28974339

[72] Eshel N, Roiser JP. Reward and punishment processing in depression. Biol Psychiatry [Internet]. 2010 Jul;68(2):118–24. Available from: 10.1016/j.biopsych.2010.01.027 20303067

[73] Gotlib IH, Joormann J. Cognition and depression: current status and future directions. Annu Rev Clin Psychol [Internet]. 2010 Mar 1 [cited 2023 Apr 4];6(1):285–312. Available from: www.annualreviews.org doi: 10.1146/annurev.clinpsy.121208.131305 20192795 PMC2845726

[74] Kircanski K, Joormann J, Gotlib IH. Cognitive aspects of depression. Wiley Interdiscip Rev Cogn Sci [Internet]. 2012 May;3(3):301–13. Available from: https://wires.onlinelibrary.wiley.com/doi/10.1002/wcs.1177 23240069 10.1002/wcs.1177 PMC3518852

[75] LeMoult J, Gotlib IH. Depression: a cognitive perspective. Clin Psychol Rev [Internet]. 2019 Apr 1 [cited 2023 Apr 4];69:51–66. Available from: https://linkinghub.elsevier.com/retrieve/pii/S0272735817303914 doi: 10.1016/j.cpr.2018.06.008 29961601 PMC11884012

[76] Kube T. If the discrepancy between expectations and actual information is too large, expectation change decreases–a replication study. J Behav Ther Exp Psychiatry [Internet]. 2023 Jun 1 [cited 2023 Mar 30];79:101831. Available from: https://linkinghub.elsevier.com/retrieve/pii/S0005791622001094 doi: 10.1016/j.jbtep.2022.101831 36521199

[77] Filipowicz A, Valadao D, Anderson B, Danckert J. Rejecting outliers: surprising changes do not always improve belief updating. Decision. 2018 Jul;5(3):165–76.

[78] Sharot T, Rollwage M, Sunstein CR, Fleming SM. Why and when beliefs change. Perspect Psychol Sci [Internet]. 2022 Aug 8;174569162210829. Available from: http://journals.sagepub.com/doi/10.1177/17456916221082967 35939828 10.1177/17456916221082967

[79] Pegg S, Ethridge P, Shields GS, Slavich GM, Weinberg A, Kujawa A. Blunted social reward responsiveness moderates the effect of lifetime social stress exposure on depressive symptoms. Front Behav Neurosci [Internet]. 2019 Aug 7;13(August):1–12. Available from: https://www.frontiersin.org/article/10.3389/fnbeh.2019.00178/full 31447659 10.3389/fnbeh.2019.00178 PMC6692494

[80] Admon R, Pizzagalli DA. Dysfunctional reward processing in depression. Curr Opin Psychol [Internet]. 2015 Aug;4:114–8. Available from: doi: 10.1016/j.copsyc.2014.12.011 26258159 PMC4525714

[81] Brockmeyer T, Holtforth MG, Pfeiffer N, Backenstrass M, Friederich HC, Bents H. Mood regulation expectancies and emotion avoidance in depression vulnerability. Pers Individ Dif [Internet]. 2012 Aug;53(3):351–4. Available from: 10.1016/j.paid.2012.03.018

[82] Joormann J, Stanton CH. Examining emotion regulation in depression: a review and future directions. Behav Res Ther [Internet]. 2016 Nov;86:35–49. Available from: doi: 10.1016/j.brat.2016.07.007 27492851

[83] Joormann J, Quinn ME. Cognitive processes and emotion regulation in depression. Depress Anxiety [Internet]. 2014 Apr;31(4):308–15. Available from: doi: 10.1002/da.22264 24668779

[84] Craske MG, Treanor M, Zbozinek TD, Vervliet B. Optimizing exposure therapy with an inhibitory retrieval approach and the OptEx Nexus. Behav Res Ther [Internet]. 2022 May;152(January):104069. Available from: doi: 10.1016/j.brat.2022.104069 35325683

[85] Huang D, Susser E, Rudolph KE, Keyes KM. Depression networks: a systematic review of the network paradigm causal assumptions. Psychol Med [Internet]. 2023/03/17. 2023 Apr 17;53(5):1665–80. Available from: https://www.cambridge.org/core/article/depression-networks-a-systematic-review-of-the-network-paradigm-causal-assumptions/C4AA6452DB0AD9796485A80E711F3F4F doi: 10.1017/S0033291723000132 36927618

