# Supplementary material for: Depressive symptoms and the processing of unexpected social feedback: Differences in surprise levels, feedback acceptance, and “immunizing” cognition
Source: PLoS One. 2024 Aug 26;19(8):e0307035. doi: 10.1371/journal.pone.0307035 (PMC11346924; doi:10.1371/journal.pone.0307035)
Supplement: S1 Appendix — (DOCX) [file pone.0307035.s001.docx]

Thoughts are considered “immunizing thoughts” if they meet these criteria:

- They **refer to** the statement in the associated video.
- They lead to a **devaluation of the positive interpersonal message** from the video, usually by being destructive or self-devaluing.

Conversely, thoughts are classified as “non-immunizing thoughts” if they meet any of these criteria:

- They have **no clear** connection to the video’s message.
- They **simply agree** with the video’s message.
- They **cannot** be clearly interpreted, or **no thoughts** were apparent at all.
- They are obviously **just a negation** of the interpersonal message, such as “he’s wrong.”

| **Interpersonal Message:** | **Examples of thoughts coded as “immunizing thoughts”** | **Examples of thoughts coded as “non-immunizing thoughts”** |
| --- | --- | --- |
| Stimulus 1: “I like you just the way you are!” | - “...it could be a lie.” - “...there might be ulterior motives.” - “...the person may not represent the majority.” | - “...that statement doesn’t apply to me.” - “...I’ve got nothing against that.” - “...it’s just his opinion.” |
| Stimulus 2: “I understand you!” | - “...many say it, but don’t mean it.” - “...nobody understands me.” - “...it’s just pity.“ | - “...it has no benefits.” - “...this statement is meaningless.” - “...she doesn’t understand me.” [simple negation] |
| Stimulus 3: “You can come to me with your problems anytime.” | - “...the person doesn’t care at all.” - “...that doesn’t help.” - “...the person isn’t honest.” | - “...I may be ashamed.” - “...lack of time.” - “...I don’t want to burden the person with my problems.” |

| Stimulus 4: “I don’t like some aspects about you.” | - “...views are subjective and one may like that aspect of themselves.” - “...she doesn’t know me well enough/long enough.” - “...not everyone has to like me.” | - “...my ego may be inflated.” - “...he doesn’t necessarily has to tell me what he don’t like about me.” - “...it's not very nice.” |
| --- | --- | --- |
| Stimulus 5: “I don’t understand you.” | - “...most people have no trouble understanding me.” - “...she doesn't want to understand me.” - “...older people often have less emotional intelligence.” | - “...that statement is not helpful.” - “...it doesn’t come across as particularly empathetic.” - “...she may not want to engage with me.” |
| Stimulus 6: “I find it tiring that you come to me so often with your problems.” | - “...she may just be having a bad day.” - “...she’s generally annoyed and that has nothing to do with me.” - “...I rarely turn to others for help with my problems.” - “...in a friendship, we sometimes do tire each other out.” | - “...it makes me feel like a burden.” - “...their statement is rude.” - “...I may not want to discuss all my problems openly.” |
