# Supplementary material for: Depressive symptoms and the processing of unexpected social feedback: Differences in surprise levels, feedback acceptance, and “immunizing” cognition
Source: PLoS One. 2024 Aug 26;19(8):e0307035. doi: 10.1371/journal.pone.0307035 (PMC11346924; doi:10.1371/journal.pone.0307035)
Supplement: S2 Appendix — (DOCX) [file pone.0307035.s002.docx]

|  | **Positive statements** (*N*  = 5) | **How surprising?** | | **How helpful?** | |
| --- | --- | --- | --- | --- | --- |
|  |  | *M* | *SD* | *M* | *SD* |
| 1. | **“I like you just the way you are!”** | **4.40** | **0.80** | **4.20** | **1.17** |
| 2. | “It's not a big deal for me if you make a mistake.” | 3.60 | 0.49 | 4.20 | 0.75 |
| 3. | “I think you’re really trying!” | 2.60 | 1.36 | 2.60 | 1.62 |
| 4. | “You don’t disappoint me just because you made a mistake.” | 3.00 | 0.63 | 4.00 | 0.89 |
| 5. | “I think we can also deal with your problems.” | 3.00 | 1.41 | 2.80 | 0.98 |
| 6. | **“You can come to me with your problems anytime.”** | **3.80** | **0.40** | **3.40** | **1.02** |
| 7. | “I'm here for you if you want to talk about your problems.” | 4.00 | 0.63 | 4.00 | 0.89 |
| 8. | “You know I’m always willing to listen to you.” | 3.60 | 0.80 | 3.80 | 0.75 |
| 9. | “I don't mind if we talk about your problems from time to time.” | 3.20 | 0.75 | 3.20 | 0.40 |
| 10. | “I think it strengthens our relationship when you speak openly with me about your problems.” | 4.00 | 0.89 | 3.40 | 1.02 |
| 11. | “I'm here for you when you need me.” | 3.00 | 0.63 | 3.80 | 0.40 |
| 12. | “Feel free to ask me for help.” | 3.20 | 0.40 | 3.40 | 0.80 |
| 13. | “You can always count on me!” | 3.20 | 0.40 | 2.60 | 1.02 |
| 14. | “I want to support you with your difficulties.” | 3.00 | 0.63 | 3.80 | 0.40 |
| 15. | “I feel like it would be better for you if we talk about your problems.” | 3.60 | 0.49 | 2.60 | 0.80 |
| 16. | “I believe that talking to me about your difficulties will make you feel better.” | 3.20 | 0.75 | 2.40 | 0.49 |
| 17. | “Talking to me about your problems will help you!” | 3.20 | 1.17 | 2.00 | 1.10 |
| 18. | “I understand your worries.” | 3.60 | 1.36 | 4.00 | 1.26 |
| 19. | **“I understand you.“** | **4.00** | **0.89** | **3.80** | **1.17** |
| 20. | “I can understand what causes you such difficulties.” | 3.80 | 1.17 | 4.00 | 1.26 |
| 21. | “I understand why you feel bad.” | 3.60 | 1.02 | 3.80 | 1.17 |
| *Note.* *N =* Sample size. All statements were rated by ten psychotherapists in training. Bold statements were finally selected because they were rated (a) above average “surprising” (cutoff: *M* = 3.46) for individuals with depression, (b) had comparatively low standard deviations, and (c) best covered the spectrum of the Depressive Expectation Scale (Kube et al., 2017). | | | | | |

|  | **Negative statements** (*N*  = 5) | **How typical?** | | **How problematic?** | |
| --- | --- | --- | --- | --- | --- |
|  |  | *M* | SD | *M* | *SD* |
| 1. | **“I don’t like some some aspects about you.”** | **3,60** | **1,02** | 2,00 | 1,10 |
| 2. | “I don't like it when you make mistakes.” | 2,60 | 1,20 | 3,20 | 1,17 |
| 3. | “You could try harder.“ | 4,20 | 0,75 | 4,20 | 0,75 |
| 4. | “I'm disappointed that you made another mistake.” | 3,80 | 1,17 | 3,60 | 1,02 |
| 5. | “I can’t deal with all of your problems.” | 4,00 | 0,63 | 3,00 | 1,10 |
| 6. | **“I find it tiring that you come to me so often with your problems.”** | **4,40** | **0,49** | **3,40** | **1,02** |
| 7. | “I can’t always be there for you when you want to talk about your problems.” | 3,80 | 0,75 | 2,80 | 1,17 |
| 8. | “You know I can't always be there to listen to you.” | 3,00 | 1,10 | 2,80 | 1,17 |
| 9. | “I find it uncomfortable that we constantly talk about your problems.” | 3,60 | 0,80 | 3,00 | 0,63 |
| 10. | “I think it strains our relationship when you speak so openly with me about your problems.” | 3,40 | 1,02 | 4,00 | 0,63 |
| 11. | “I can’t always be there for you when you need me.” | 3,00 | 0,89 | 3,00 | 0,63 |
| 12. | “Please turn to someone else if you need help.” | 2,80 | 0,40 | 2,40 | 1,02 |
| 13. | “Stop relying on me all the time!” | 2,60 | 0,49 | 2,40 | 0,49 |
| 14. | “I don’t want to have to constantly support you with your difficulties.” | 3,00 | 0,89 | 3,00 | 1,10 |
| 15. | “I don’t think like talking about your problems would make you feel better.” | 2,00 | 1,26 | 2,80 | 1,17 |
| 16. | “I don’t believe that talking to me about your difficulties will make you feel better.” | 2,00 | 0,89 | 2,40 | 1,02 |
| 17. | “Talking to me about your problems won't help you!” | 2,00 | 1,55 | 2,80 | 1,17 |
| 18. | “I can’t understand your worries.” | 4,20 | 0,75 | 3,40 | 1,02 |
| 19. | **„I don’t understand you.“** | **4,40** | **0,49** | **3,60** | **0,49** |
| 20. | “I don’t understand what causes you such difficulties.” | 4,40 | 0,49 | 3,80 | 0,98 |
| 21. | “I don’t understand why you feel so bad.” | 4,20 | 0,40 | 3,80 | 0,75 |
| *Note.* *N =* Sample size. All statements were rated by ten psychotherapists in training. Bold statements were finally selected, as they were rated (a) above-average “typical” (cutoff: *M* = 3.38) and “problematic” for individuals with depression, (b) had comparatively low standard deviations, and (c) best covered the spectrum of the Depressive Expectation Scale (Kube et al., 2017). | | | | | |
