# Supplementary material for: Depressive symptoms and the processing of unexpected social feedback: Differences in surprise levels, feedback acceptance, and “immunizing” cognition
Source: PLoS One. 2024 Aug 26;19(8):e0307035. doi: 10.1371/journal.pone.0307035 (PMC11346924; doi:10.1371/journal.pone.0307035)
Supplement: S3 Appendix — (DOCX) [file pone.0307035.s003.docx]

| **Pilot sample characteristics** | **Audio**  (*N* = 9) | **Video**  (*N* = 11) |
| --- | --- | --- |
| **Gender** male, *N* (%) female, *N* (%)  diverse, *N* (%) | 6 (66.67) 3 (33.33) 0 (0.00) | 9 (81.82) 2 (18.18) 0 (0.00) |
| **Citizenship** German, *N* (%) mixed, *N* (%) other, *N* (%) | 9 (100.00) 0 (0.00) 0 (0.00) | 10 (90.91) 1 (9.09) 0 (0.00) |
| **Legal status** unmarried, *N* (%) married, *N* (%) divorced, *N* (%) widowed, *N* (%) registered partnership, *N* (%) | 5 (55.56) 3 (33.33) 0 (0.00) 1 (11.11) 0 (0.00) | 7 (63.64) 4 (36.36) 0 (0.00) 0 (0.00) 0 (0.00) |
| **Relationship Status** single, *N* (%) partnership, *N* (%) | 3 (33.33) 6 (66.67) | 6 (54.55) 5 (45.45) |
| **Education** primary school diploma, *N* (%) secondary school diploma, *N* (%) high school diploma, *N* (%) | 0 (0.00) 1 (11.11) 8 (88.89) | 0 (0.00) 2 (18.18) 9 (81.82) |
| **Vocational training** none/not completed, *N* (%) completed, *N* (%) Bachelor’s degree, *N* (%) Master’s degree, *N* (%) PhD, *N* (%) | 2 (22.22) 2 (22.22) 2 (22.22) 3 (33.33) 0 (0.00) | 0 (0.00) 6 (54.55) 3 (27.27) 2 (18.18) 0 (0.00) |
| **Employment** full-time, *N* (%) part-time, *N* (%) not employed, *N* (%) | 7 (77.78) 1 (11.11) 1 (11.11) | 10 (90.91) 0 (0.00) 1 (9.09) |
| **Migration** immigrant, *N* (%) no immigrant, *N* (%) | 0 (0.00) 9 (100.00) | 1 (9.09) 10 (90.91) |
| **Age**, *M (SD)* | 30.56 (9.68) | 38.64 (9.16) |
| **Weekly working time**, *M (SD)* | 34.22 (13.28) | 36.09 (12.02) |
| **Household members**, *M (SD)* | 3.22 (1.64) | 2.55 (1.92) |
| **Net household income**, *M (SD)* | 3506.17 (2572.05) | 3181.82 (1296.78) |
| **BSI-18^a^** overall depression, *M (SD)* anxiety, *M (SD)* somatization, *M (SD)* | 8.44 (9.28) 2.78 (3.90) 3.56 (3.28) 2.11 (2.93) | 5.09 (3.24) 1.46 (1.92) 2.27 (1.79) 1.36 (1.57) |
| **Surprise** after positive feedback, *M (SD)* after negative feedback, *M (SD)* | 2.67 (1.01) 3.67 (0.75) | 1.85 (0.69) 3.93 (1.02) |
| **Feedback-acceptance** after positive feedback, *M (SD)* after negative feedback, *M (SD)* | 4.85 (1.09) 3.48 (0.77) | 5.24 (0.60) 3.77 (0.89) |
| **Immunizing cognition**  after positive feedback, *M (SD)*  after negative feedback, *M (SD)* | 1.58 (1.05) 2.10 (0.74) | 1.51 (0.83) 1.66 (1.02) |
| **Distress due to participation**, *M (SD)* | 2.15 (1.00) | 2.00 (1.08) |
| *Note.* *N* = Sample size, *M =* Mean, *SD* = Standard Deviation, MDD = Major Depressive Disorder.  ^a^ For reasons of comparability, we report mean sum scores here although we used mean composite scores in our main analyses. | | |

Inspecting the immunizing thoughts generated by the participants in the pilot study, as well as their answers in the follow-up survey, revealed that the instructions were comprehensible and that the experimental procedure resulted in usable data.

We conducted two single-factor MANCOVAs separately for positive (Wilk’s λ = 0.76, *F* (3, 15) = 1.59, *p* = .234) and negative social feedback (Wilk’s λ = 0.89, *F* (3, 13) = 0.53, *p* = .67) with *Surprise*, *Feedback Acceptance,* and *Immunizing Cognition* as dependent variables (DVs) and *Depressive Symptoms* as covariate. Since neither analysis yielded any group differences, we decided to use the video stimuli in the main study due to descriptive comparisons (e.g., higher differences in the DVs *Surprise After Social Feedback* and *Feedback-Acceptance* in response to positive feedback compared to negative feedback).
