# Supplementary material for: Depressive symptoms and the processing of unexpected social feedback: Differences in surprise levels, feedback acceptance, and “immunizing” cognition
Source: PLoS One. 2024 Aug 26;19(8):e0307035. doi: 10.1371/journal.pone.0307035 (PMC11346924; doi:10.1371/journal.pone.0307035)
Supplement: S4 Appendix — The red graph corresponds to the edge estimate in the sample, the black graph to the mean edge estimate in the bootstrapped samples and the gray area to the 95% confidence interval band from the bootstraps edge weights. (PPTX) [file pone.0307035.s004.pptx]

## Slide 1
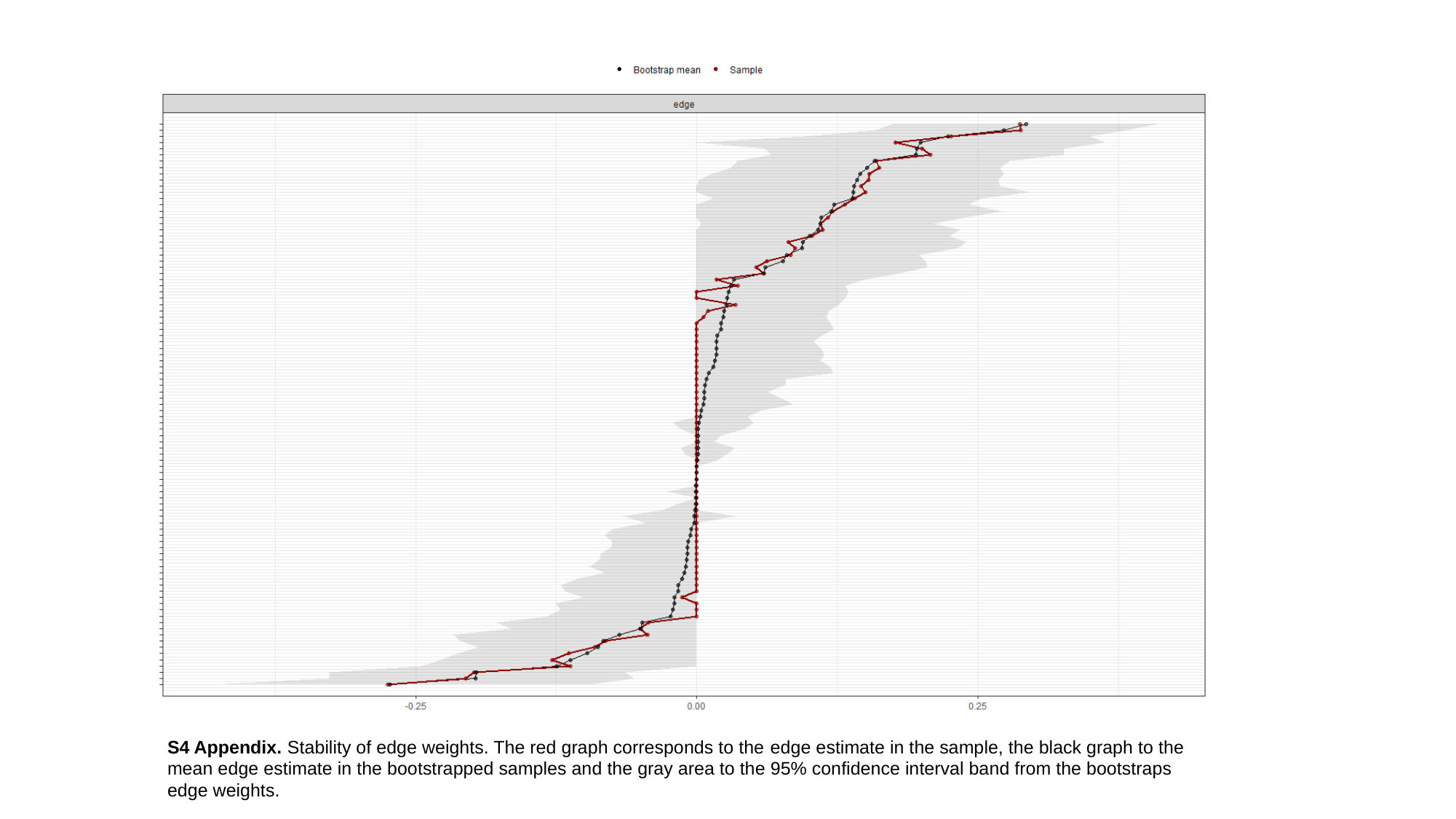

S4 Appendix. Stability of edge weights. The red graph corresponds to the edge estimate in the sample, the black graph to the mean edge estimate in the bootstrapped samples and the gray area to the 95% confidence interval band from the bootstraps edge weights.
