# Supplementary material for: Depressive symptoms and the processing of unexpected social feedback: Differences in surprise levels, feedback acceptance, and “immunizing” cognition
Source: PLoS One. 2024 Aug 26;19(8):e0307035. doi: 10.1371/journal.pone.0307035 (PMC11346924; doi:10.1371/journal.pone.0307035)
Supplement: S5 Appendix — (PPTX) [file pone.0307035.s005.pptx]

## Slide 1
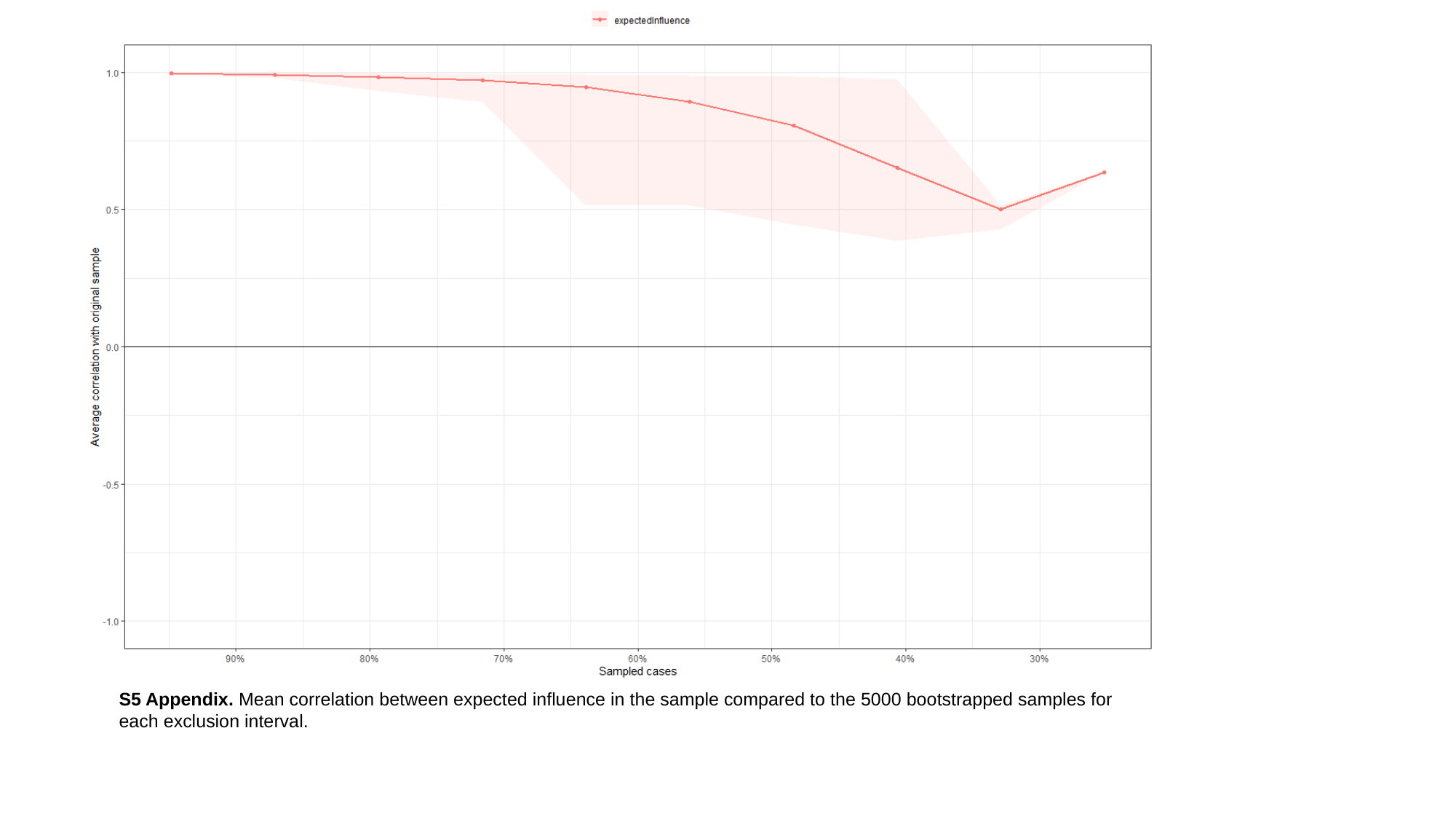

S5 Appendix. Mean correlation between expected influence in the sample compared to the 5000 bootstrapped samples for each exclusion interval.
